# Characterization of NADPH Oxidase Expression and Activity in Acute Myeloid Leukemia Cell Lines: A Correlation with the Differentiation Status

**DOI:** 10.3390/antiox10030498

**Published:** 2021-03-23

**Authors:** Hassan Dakik, Maya El Dor, Joan Leclerc, Farah Kouzi, Ali Nehme, Margaux Deynoux, Christelle Debeissat, Georges Khamis, Elfi Ducrocq, Aida Ibrik, Marie-José Stasia, Houssam Raad, Hamid Reza Rezvani, Fabrice Gouilleux, Kazem Zibara, Olivier Herault, Frédéric Mazurier

**Affiliations:** 1 University of Tours EA 7501, CNRS ERL 7001, LNOx Team, F-37032 Tours, France; maya.el-dor@etu.univ-tours.fr (M.E.D.); leclerc_joan@yahoo.fr (J.L.); farah.kouzi@etu.univ-tours.fr (F.K.); ali.nehme2@mcgill.ca (A.N.); margaux.deynoux@etu.univ-tours.fr (M.D.); christelle.debeissat@u-bordeaux.fr (C.D.); georges.khamis@univ-tours.fr (G.K.); elfi.ducrocq@univ-tours.fr (E.D.); fabrice.gouilleux@univ-tours.fr (F.G.); olivier.herault@univ-tours.fr (O.H.); 2 PRASE, Lebanese University, 6573/14 Beirut, Lebanon; aida.ibrik@ul.edu.lb; 3 University Grenoble Alpes, CEA, CNRS, IBS, F-38044 Grenoble, France; MJStasia@chu-grenoble.fr; 4 CDiReC, Pôle Biologie, CHU de Grenoble, F-38043 Grenoble, France; 5 University of Bordeaux, INSERM U1035, F-33000 Bordeaux, France; houssam.raad@ul.edu.lb (H.R.); hamid-reza.rezvani@u-bordeaux.fr (H.R.R.); 6 Biology Department, Faculty of Sciences-I, Lebanese University, 90656 Beirut, Lebanon; 7 CNRS GDR 3697 MicroNiT, F-37032 Tours, France; 8 CHRU de Tours, Service d’Hématologie Biologique, F-37000 Tours, France

**Keywords:** leukemia, AML, transcriptomics, NADPH oxidase

## Abstract

In acute myeloid leukemia (AML), a low level of reactive oxygen species (ROS) is associated with leukemic stem cell (LSC) quiescence, whereas a high level promotes blast proliferation. ROS homeostasis relies on a tightly-regulated balance between the antioxidant and oxidant systems. Among the oxidants, NADPH oxidases (NOX) generate ROS as a physiological function. Although it has been reported in AML initiation and development, the contribution of NOX to the ROS production in AML remains to be clarified. The aim of this study was to investigate the NOX expression and function in AML, and to examine the role of NOX in blast proliferation and differentiation. First, we interrogated the NOX expression in primary cells from public datasets, and investigated their association with prognostic markers. Next, we explored the NOX expression and activity in AML cell lines, and studied the impact of NOX knockdown on cell proliferation and differentiation. We found that NOX2 is ubiquitously expressed in AML blasts, and particularly in cells from the myelomonocytic (M4) and monocytic (M5) stages; however, it is less expressed in LSCs and in relapsed AML. This is consistent with an increased expression throughout normal hematopoietic differentiation, and is reflected in AML cell lines. Nevertheless, no endogenous NOX activity could be detected in the absence of PMA stimulation. Furthermore, *CYBB* knockdown, although hampering induced NOX2 activity, did not affect the proliferation and differentiation of THP-1 and HL-60 cells. In summary, our data suggest that NOX2 is a marker of AML blast differentiation, while AML cell lines lack any NOX2 endogenous activity.

## 1. Introduction

Over the last few decades, oxidative stress has been reported in solid tumors as well as in hematopoietic malignancies [1,2] including acute myeloid leukemia (AML) [3]. Indeed, reactive oxygen species (ROS) are key messengers in cell signaling [4] that can modulate the activity of redox-sensitive proteins, and can influence cellular processes encompassing metabolism, survival, proliferation, and apoptosis [5]. In AML, a low level of ROS has been associated with the self-renewal of leukemic stem cells (LSC) and aggressiveness, whereas a moderate level was linked to blast proliferation [6,7,8]. Redox homeostasis is the result of a tightly-regulated balance between ROS production and detoxification. Although the antioxidant system is extensively studied in AML, the oxidant system remains poorly understood.

Under physiological conditions, the mitochondrial respiratory chain, NO synthases (NOS), xanthine dehydrogenase/oxidase (XDH), and NADPH oxidases (NOX) are major contributors to ROS production [9]. Unlike most oxidative enzymes that generate ROS as by-products, NOX produce ROS as main function [10]. The NOX family comprises seven members, NOX1–5 and DUOX1–2, which can transfer electrons across biological membranes from NADPH to molecular oxygen (O_2_), catalyzing its transformation into superoxide anion (O_2_^−^) or hydrogen peroxide (H_2_O_2_) [11,12]. Indeed, NOX activity has been widely reported in solid tumors [13,14,15,16,17,18], and was found to promote tumorigenesis by regulating cell survival, division, and angiogenesis [19,20,21]. This drew particular attention to NOX as potential targets in cancer [22,23]. Likewise, NOX were shown to participate in AML transformation [24], proliferation [6,25], migration [25], differentiation blockage [26,27], and in the self-renewal of leukemic stem cells [27]. However, the characterization of the NOX family in myeloid leukemia remains limited, and studies had mainly focused on NOX2 and NOX4 complexes [6,8,24,25,28]. Besides this, the studies mostly relied on gene expression and NOX inhibition, by chemicals or knockdown, and the enzymatic activity of NOX has not been accurately investigated. Therefore, the contribution of NOX to ROS production in AML needs further investigation.

In this work, we performed an extensive transcriptional analysis of all of the NOX subunits in AML, using public transcriptomic datasets and a large panel of 24 AML cell lines. We also evaluated the prognostic value of NOX genes in three AML cohorts, and examined whether NOX activity contributes to AML proliferation and differentiation-blockage using a knockdown strategy in AML cell lines.

## 2. Materials and Methods

### 2.1. Analysis of the Public Datasets

Transcriptomics: raw CEL files from the AML microarray datasets GSE6891 and GSE10358 (Affymetrix GeneChip Human Genome U133 Plus 2.0) were downloaded from the GEO Omnibus (https://www.ncbi.nlm.nih.gov/geo/ (last accessed on 18 March 2021)). The datasets were subjected to background-correction and Robust Multichip Average (RMA) normalization using BioConductor’s Affy package [29] in the R environment [30]. Preprocessed microarray datasets of normal hematopoiesis (GSE24759) [31], paired diagnosis and relapse AML samples (GSE65625) [32], and functionally annotated leukemic stem cell enriched (LSC^+^) and depleted (LSC^−^) AML populations (GSE76009) [33] were also downloaded from the GEO Omnibus. For each gene, the probe set with the highest average expression across the samples was used for the analysis. The AML dataset for the Cancer Genome Atlas TCGA was acquired using cBioPortal’s cgdsr package in R environment (https://www.cbioportal.org/) (last accessed on 18 March 2021).

Proteomics: the MaxQuant output files for the PXD019785 dataset were downloaded from the PRIDE database [34,35]. The data processing and normalization were performed using the DEP package in the R environment [36].

Boxplots and principal component analysis (PCA) plots were created in the R environment using the ggplot2 package [37]. Hierarchical clustering and correlation plots were generated in R using the ComplexHeatmap [38] and corrplot [39] packages. For the cell lines and the GSE24759 dataset, the heatmaps were created using the Clustvis web tool [40].

### 2.2. Survival Analysis

Overall survival (OS) was defined as the time from the AML diagnosis until death, and the patients who were alive at last follow-up were censored. Event-free survival (EFS) was defined as the time from the diagnosis until an event occurred (induction failure, relapse, or death), and the patients with no events at their last follow-up were censored. Cox proportional hazard (CPH) regression was used to perform the univariate and multivariate analyses [41]. The violation of the proportional hazards assumption was examined using Schoenfeld residuals [42]. For the TCGA dataset, age violated the proportional hazard’s assumption, and was therefore used as the stratifier, as took place in other research [33]. Wald’s test was used to evaluate the significance of the individual regression coefficients. The survival analysis was performed in the R environment using the survival package [43].

### 2.3. Cell Lines and Culture

Twenty-Four human myeloid leukaemia cell lines were purchased from DSMZ (German Collection of Microorganisms & Cell Cultures, Braunschweig, Germany), and cultured in the recommended media (Table S1) at 37 °C in humidified air and 5% CO_2_. The cells were harvested in their exponential growth phase. Cytarabine-resistant cell lines were established, as described previously [44]. The cells were first cultured in the presence of 0.1 µM of cytarabine (Ara-C), and the dose was progressively increased by 0.1 µM with every passage, until a resistance to 1 µM of Ara-C was reached.

### 2.4. RNA Extraction and Real-Time Reverse Transcription Quantitative PCR (RT-qPCR) Analysis

The cells were washed twice with cold Phosphate Buffer Saline (PBS) before pelleting. The total cellular RNA was extracted using a Maxwell RNA purification kit (Promega, Charbonnières-les-Bains, France), and was quantified using a NanoDrop Lite spectrophotometer. The RNA purity was analyzed using Agilent 2100 Bioanalyzer (Agilent Technologies, Les Ulis, France). Five micrograms of RNA were reverse-transcribed using the SuperScript^®^ VILO TM cDNA Synthesis kit (Invitrogen, Paris, France). For most of the genes, primer (Eurogentec, Liége, Belgium) validation was performed on Stratagene’s cDNA mix (Agilent Technologies, Les Ulys, France). For the NOX1, NOX3, and NOXO1 primers, the validation was performed on plasmids carrying the corresponding cDNA; the pcDNA3.1-hNox1 (Addgene, Watertown, MA, USA) plasmid #58344), pcDNA3.1-hNoxo1 (Addgene plasmid #58530) and pcDNA3.1-hNox3 (Addgene plasmid #58341) were gifts from Botond Banfi and Karl-Heinz Krause. Universal ProbeLibrary probes were purchased from Roche (Meylan, France). The sequences of primers and probes are documented in Table S2.

The RT-qPCR reactions were performed on 20 ng of cDNA using a LightCycler^®^ 480 Probes Master (Roche), as described previously [45]. The samples were subjected to an initial denaturation step (5 min, 95 °C), followed by 45 PCR cycles (10 s, 95 °C, then 30 s, 60 °C) and a final cooling step (40 °C, 30 s). Triplicates of each sample were analyzed using the cycle threshold (Ct) values determined by the LightCycler^®^ 480 software. The geometric Ct mean of human *ACTB*, *YWHAZ* and *RPL13A* genes was used as the endogenous control, in order to calculate the expression of the target genes: ΔCT = “Ct gene” − “Ct reference geomean”.

### 2.5. Western Blot

Radioimmunoprecipitation assay (RIPA) buffer (50 mM Tris-HCl pH7.5, 0.1% SDS, 1% Triton, 150 mM NaCl, 1 mM EDTA) was used for the extraction of the NCF1 (Merck Millipore, Fontenay-sous-Bois, France; Ref. 07-001, 1/3000 dilution), NCF2 (Merck Millipore, Ref. 07-002, 1/3000 dilution), and NCF4 (EMD Millipore, Ref. 07-503, 1/3000 dilution) proteins, while Laemmli buffer (62.5 mM Tris-HCl pH 6.8, 2% SDS, 10% glycerol, 5% β-mercaptoethanol, 0.005% bromophenol blue) was used for the extraction of the CYBA protein (Abcam, Paris, France; Ref. ab80896, 1/500 dilution). The RIPA buffer was supplemented with a protease inhibitor cocktail (Roche) and a phosphatase inhibitor cocktail (Thermo Fisher scientific, Villebon-sur-Yvette, France) according to the suppliers’ instructions, and 1 mM of phenylmethylsulfonyl fluoride (PMSF). The extracted proteins were quantified using the bicinchoninic acid method. The lysates were then diluted to 1 µg/µL in Laemmli loading buffer and heated at 95 °C for 5 min before loading. Up to 40 µg of the proteins were loaded per well, and SDS-page was performed using 4–15% gradient polyacrylamide gels (BioRad, Marnes-la-Coquette, France). The proteins were transferred onto 0.2 µm nitrocellulose membranes (BioRad), and were blocked for 1 h at room temperature in Tris-buffered saline (TBS)-Tween (0.2%) with 5% milk. The membranes were then incubated overnight at 4 °C with the appropriate dilutions of the primary antibodies. The horseradish peroxidase (HRP)-conjugated secondary anti-mouse and anti-rabbit antibodies were purchased from Vectorlabs (Peterborough, UK), and the band detection was performed using an Amersham enhanced chemiluminescence (ECL) detection kit (GE Healthcare Life Sciences, Velizy-Villacoublay, France). The protein quantification was performed using ImageJ software.

### 2.6. Enzymatic Activity Assay

The NOX activity was measured as previously described, with some modifications [14]. The cells were washed in PBS, and were seeded in 96-well plates at 2 × 10^5^ cells per well in an isotonic glucose solution (300 mM) containing luminol (10 µM) (Sigma-Aldrich, Lyon, France) and horseradish peroxidase (10 U/well) (Sigma-Aldrich). Phorbol 12-myristate 13-acetate (PMA; Sigma-Aldrich) was added at a final concentration of 100 nM prior to the measurement. The luminescence was measured every minute for 2 h at 25 °C using a ClarioStar microplate reader (BMG Labtech, Champigny-sur-Marne, France). The area under the curve was calculated via ClarioStar Data Analysis software from the average of replicates of blank-corrected data. Diphenyleneiodonium chloride (DPI; Sigma-Aldrich) and VAS3947 (Merck Millipore), both at 20 µM, were used to block the NOX activity. Dimethyl Sulfoxide (DMSO; Sigma-Aldrich) was used as the vehicle control.

### 2.7. Lentiviral Production and Transduction

Lentiviral particles expressing shRNA sequences against luciferase mRNA (*shLUC*: CACGTACGCGGAATACTTCGA) or *CYBB* (*shCYBB*: TGCCTGAATTTCAACTGCATG), and harboring puromycin N-acetyltransferase (PAC) as a reporter gene, were produced as previously described [46]. The HL-60 and THP-1 cell lines were transduced with a multiplicity of infection (MOI) of five. The transduced cells were subjected to a 6-day selection period with 1 µg/mL of puromycin prior to the experiments.

### 2.8. Cell Differentiation

The differentiation of THP-1 cells was induced by PMA (100 nM), while that of the HL-60 cells was induced by a mix of all-trans retinoic acid (ATRA, 100 nM) and DMSO (1.25%), as previously described [47]. In brief, 2 × 10^5^ cells were cultured in 3 mL media in 6-well plates, either in the presence or absence of the differentiation agents, and were incubated at 37 °C for 96 h. The cell differentiation was checked by flow cytometry using anti-CD11b-PE antibody (Becton Dickinson, Le Pont de Claix, France). A Phycoerythrin (PE)-coupled mouse IgG1κ was used as the isotype control (Becton Dickinson). The data acquisition was performed on a C6 Accuri^®^ flow cytometer, and the analysis was performed using FlowJo^®^ software (Becton Dickinson).

### 2.9. Cell Proliferation

Cell counting: transduced cells were seeded in 3 mL media at a density of 2 × 10^5^ cells/well in 6-well plates, and were incubated at 37 °C for 72 h. The cell numbers were counted on day 3 using a trypan blue exclusion assay.

Resazurin assay: transduced cells were seeded in 200 µL media at a density of 5 × 10^4^ cells/well in 96 well-plates, and were incubated at 37 °C for 72 h. The cell proliferation was followed at days 0 and 3 using a resazurin reduction assay. At both timepoints, resazurin (0.1 mg/mL) (Sigma-Aldrich) was added at a volume of 20 µL per well, and the plates were incubated for 4 h at 37 °C. The fluorescence (λ_ex_ = 529.5–19 nm, λ_em_ = 582–36 nm) was measured using a ClarioStar microplate reader. The blank corrected signals were calculated using ClarioStar Data Analysis software, and normalization was performed with respect to day 0 for each condition.

### 2.10. Statistical Analyses

For the cell lines, the results are expressed as mean ± SEM of three independent experiments. R software was used to construct the figures and perform the statistical analyses.

## 3. Results

### 3.1. NOX2 Complex Components Are Ubiquitously Expressed in AML, but Show Lower Expression in the t(8;21) and t(15;17) Subgroups

**Figure 1:**
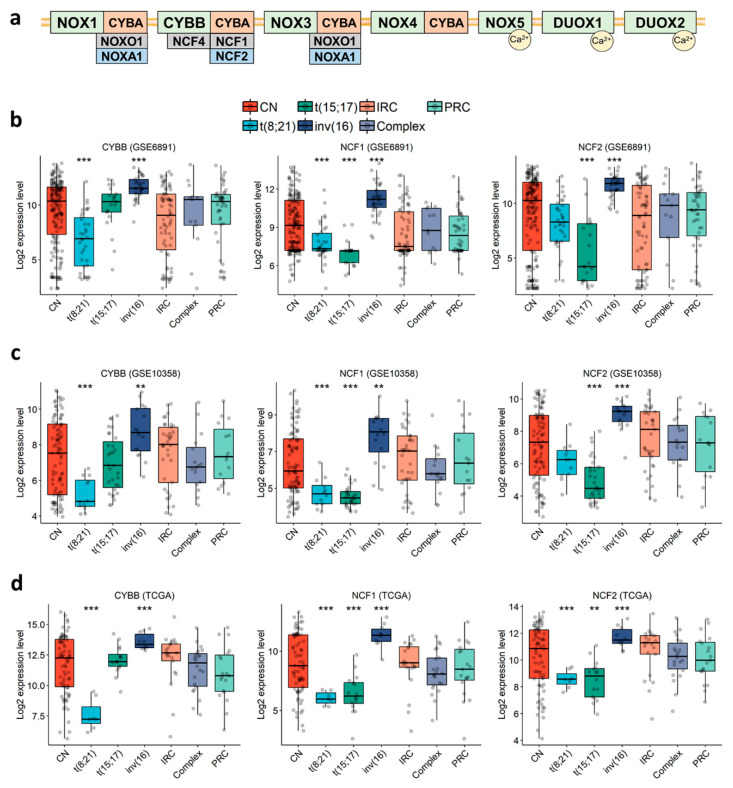
Expression profile of genes coding for CYBB, NCF1, and NCF2 in primary AML, according to cytogenetic abnormalities. (**a**) Schematic representation of the seven NOX complexes (NOX1-5 and DUOX1-2) and their 13 subunits. The analysis was performed in the (**b**) GSE6891, (**c**) GSE10358, (**d**) TCGA datasets. Student’s t-test followed by Benjamini-Hochberg (BH)-adjustment was used to compare each karyotype to all of the others combined. **: *p* < 0.01; ***: *p* < 0.001. CN: cytogenetically normal. IRC: intermediate risk cytogenetics. PRC: poor risk cytogenetics.

In order to characterize the expression profile of NOX-coding genes in AML cells (Figure 1a), we examined three public expression datasets of primary AML: GSE6891 (microarray, N = 443) [48], GSE10358 (microarray, N = 223) [49], and TCGA (RNA-seq, N = 173) [50]. The data showed that genes coding for the subunits of NOX2 complex (*CYBB*, *NCF1*, *NCF2*, *NCF4* and *CYBA*) were highly expressed in most AML samples, whereas genes coding for NOX1, NOX3-5, DUOX1-2, NOXA1 and NOXO1 were either weakly expressed or absent (Table S3). Besides this, three genes (*CYBB*, *NCF1* and *NCF2*) coding for NOX2 complex subunits were constantly deregulated across the three datasets (Figure 1b–d and Table S4). Each of these genes was deregulated in at least two of the three favorable cytogenetic groups—t(8;21), t(15;17) and inv(16)—compared to the other groups. Remarkably, they were also highly expressed in inv(16) compared to the other groups (Figure 1b–d and Table S4. Fold Change (FC) > 1.5 with *p* < 0.002). In addition, *CYBB* had the lowest expression in the t(8;21) group (Figure 1b–d and Table S4. FC < −2.1 with *p* < 0.001), and *NCF1* had the lowest expression in the t(8;21) and t(15;17) groups (Figure 1b–d and Table S4. FC < −1.2 and −1.8 for t(8;21) and t(15;17), respectively, with *p* < 0.001), whereas *NCF2* had its lowest expression in the t(15;17) group (Figure 1b–d and Table S4. FC < −2.4 with *p* < 0.001). These findings indicate that NOX2 is the most expressed NOX complex in AML, but it shows lower expression within the t(8;21) and t(15;17) subgroups.

### 3.2. NOX-Coding Genes Are not Prognostic in AML

Because gene expression varies between subtypes, we aimed to examine whether NOX-coding genes may have a prognostic value in AML. Thus, we performed Cox regression analysis across the three AML datasets. The results showed that none of the 13 NOX-coding genes were consistently prognostic in all of the datasets, either in univariate analysis (Table S5) or after adjustment for age and cytogenetic abnormalities (Table S6). However, *NCF4* expression correlated with a bad prognosis for EFS in the GSE10358 and TCGA datasets (Tables S5 and S6). We also examined the correlation between NOX expression and somatic mutations within the cytogenetically-normal AML subset, but found no correlation with *NPM1/FLT3ITD* status (Table S7). We also found that none of the genes was consistently prognostic within the cytogenetically-normal AML, subset either in the univariate analysis or after adjustment for age and *NPM1/FLT3ITD* status (Tables S8 and S9).

### 3.3. Subunits of the NOX2 Complex Are Highly Expressed among M4 and M5 AML Primary Blasts

**Figure 2:**
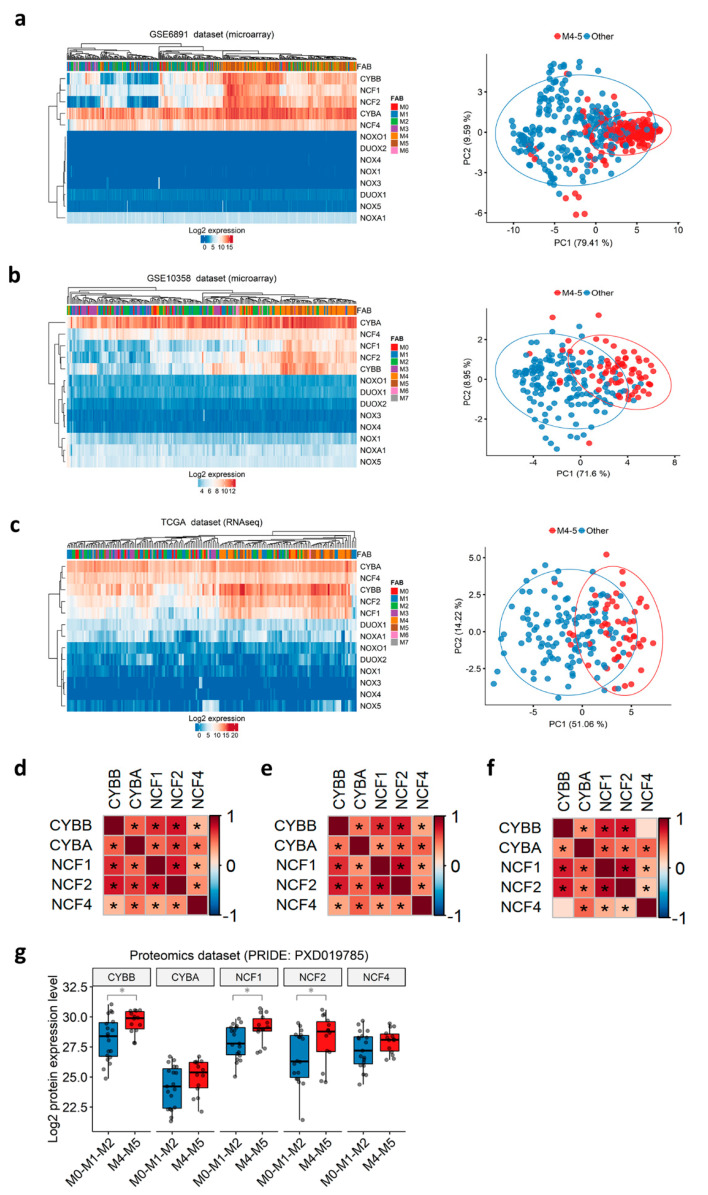
NOX expression profile in primary AML, according to FAB subtypes. (**a**–**c**) Heatmap (left) and principal component analysis (PCA) plots (right) depicting the expression of NOX genes in primary AML samples from the GSE6891, GSE10358, and TCGA datasets, respectively. In the heatmap, the data are shown as log2-expression values. Both the rows and columns are clustered using Euclidean distance and average linkage. In the PCA plot, the first two principal components explaining most of the variability between the samples are shown. Together, PC1 and PC2 explain 89% of the total variance in the NOX expressions between the samples. The samples from the M4/M5 subtypes are colored in red, while the other subtypes are in blue. The prediction ellipses are such that, with probability 0.95, a new observation from the same group will fall inside the ellipse. (**d**–**f**) Correlations between the genes coding for the NOX2 complex in the GSE6891, GSE10358, and TCGA datasets, respectively. The pairwise correlation between the genes was estimated using Pearson’s correlation coefficient. The positive coefficients (in red) indicate a positive correlation, while the negative coefficients (in blue) indicate a negative correlation. The BH-adjusted *p*-values are also reported, and an asterisk (*) indicates a significant correlation (*p* < 0.05). (**g**) The NOX proteomic profile in primary AML according to the FAB subtypes in PRIDE’s PXD019785 dataset (N = 33). The pairwise comparisons between the M4–M5 and M0-M1-M2 groups were performed using Student’s *t*-test (*: *p* < 0.05).

Before the European LeukemiaNet classification, AML patients were historically stratified into eight French-American-British (FAB) subtypes (M0–M7), according to the differentiation orientation and degree of maturation of the blasts [51,52]. We noticed that the t(8;21) group, in which NOX2-coding genes are down-regulated, is correlated with the M2 FAB subtype (71.4–85.7%), whereas the inv(16) group, in which NOX2-coding genes are up-regulated, is correlated with the more mature M4 subtype (70–93.8%) in all three datasets (Table S10). This respective correlation between the two Core Binding Factor (CBF) translocations and M2/M4 subtypes has been previously reported [53]. We thus speculated that the NOX expression profile in AML could be associated with the commitment of AML blasts and their stage of blockage. Therefore, we investigated the expression profile of all NOX-coding genes according to the FAB subtypes. The expression of genes coding for the NOX2 complex was the highest in the more mature myelomonocytic (M4) and monocytic (M5) subtypes, as shown by hierarchical clustering and principal component analysis (Figure 2a–c). Genes coding for other complexes are virtually undetectable, as indicated in Table S3 (Figure 2a–c). Furthermore, the expression of NOX2 complex genes showed positive inter-correlation in all of the datasets (Figure 2d–f).

The transcriptomic findings were validated at the protein level in a proteomics dataset of 33 AML samples (PXD019785) [35]. As expected, none of the NOX1, NOX3–5, or DUOX proteins was detected in this dataset. In contrast, all of the proteins from the NOX2 complex were detected, and the expression of CYBB, NCF1, and NCF2 proteins was increased in the M4–M5 primary AML compared to more immature FAB groups (Figure 2g).

Together, the data indicate that the expression of NOX2 complex genes in AML blasts might be dependent on the stage of differentiation blockage, and that these genes are tightly co-regulated.

### 3.4. The Expression of NOX2 Complex Subunits Increases throughout Normal Myeloid Differentiation

**Figure 3:**
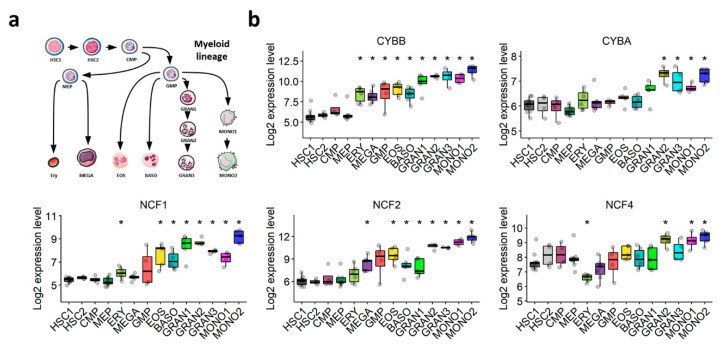
Expression profile of genes coding for NOX2 subunits throughout normal myeloid differentiation (GSE24759). (**a**) Schematic representation of the myeloid differentiation hierarchy depicting the analyzed populations. (**b**) Boxplots showing the expression of genes coding for the subunits of the NOX2 complex in myeloid subsets. Wilcoxon’s rank-sum test followed by BH-adjustment was used for the pairwise comparisons between the HSC1 population and the other populations. *: *p* < 0.05. HSC1: CD133^+^CD34^dim^ HSCs; HSC2: CD38^−^CD34^+^ HSCs; CMP: common myeloid progenitor; GMP: granulocyte/monocyte progenitor; MEP: megakaryocyte/erythroid progenitor; GRAN1: colony-forming unit-granulocyte; GRAN2: granulocyte (neutrophilic metamyelocyte); GRAN3: granulocyte (neutrophil); EOS: eosinophil; BASO: basophil; MONO1: colony-forming unit-monocyte; MONO2: monocyte, Ery: CD34^−^ CD71^−^ GlyA^+^ erythroid; MEGA: megakaryocyte.

The NOX2 complex was first discovered in the oxidative burst of phagocytes. We postulated that its expression would increase throughout normal myeloid differentiation. The transcriptomic exploration of a comprehensive dataset of normal hematopoiesis (GSE24759) showed that the expression of NOX2 complex subunits is weak in HSCs, but increases gradually during normal granulocytic and monocytic differentiation (Figure 3a,b). This corroborates our hypothesis that the expression of genes coding for components of the NOX2 complex in AML blasts may depend on their differentiation status, rather than being related to AML malignancy.

### 3.5. NOX2 Complex Subunits Are Depleted in LSCs and at Relapse

**Figure 4:**
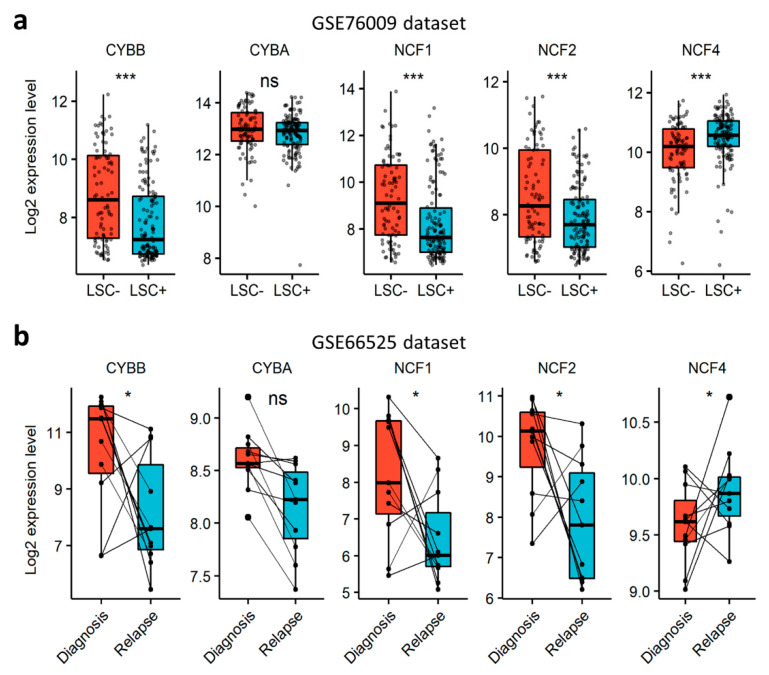
Expression profile of genes coding for NOX2 subunits, according to AML stemness and resistance to therapy. (**a**) The expression profile of genes coding for NOX2 subunits in an AML-engrafting leukemic stem cell enriched (LSC^+^) population compared to a non-engrafting LSC depleted (LSC^−^) population from the GSE76009 dataset [33]. Student’s *t*-test, followed by BH-adjustment, was used for the pairwise comparisons between the LSC^+^ and LSC^−^ groups. (**b**) The expression profile of genes coding for NOX2 subunits in AML patients at relapse compared to diagnosis from the GSE66525 dataset [32]. Wilcoxon’s signed-rank test was used for the pairwise comparisons between relapse and diagnosis. ns: not significant, *: *p* < 0.05; ***: *p* < 0.001.

In order to verify whether NOX can contribute to AML aggressiveness, we investigated the expression of NOX-coding genes in LSCs and in relapsed AML cells. First, we explored the NOX-expression profile in functionally-defined LSCs using a dataset (GSE76009) of mouse-engrafting primary human AML stem cells (LSC^+^) and non-engrafting LSC-depleted (LSC^−^) cells [33]. The expression of the catalytical subunits was examined according to the expression status of the CD34 and CD38 cell surface markers. *CYBB* was the most expressed gene, compared to *NOX1*, *NOX3-5* and *DUOX1/2* (Figure S1a). The expression of *CYBB* was weak in CD34^+^CD38^−^ cells, which are enriched in LSC activity, but increased with the loss of the CD34 marker in more committed cells. The expression of NOX2 complex regulatory subunits, save for *NCF4*, was strongly induced in more differentiated CD34^−^CD38^+^ cells (Figure S1b). In line with this, NOX2 complex genes, except *NCF4*, were downregulated in LSC^+^ compared to LSC^−^ cells (Figure 4a), indicating that cells with LSC properties have a lower expression of genes coding for the components of the NOX2 complex compared to more committed cells. Next, we examined the expression of NOX2 complex genes in AML samples from the GSE66525 dataset at relapse and diagnosis [32]. Consistent with the observation in LSCs, we found a global decrease in the expression of NOX2 genes at relapse compared to diagnosis, except for *NCF4* (Figure 4b). Interestingly, an LSC signature has been reported in AML blasts at relapse for this dataset [32]. In order to address the negative correlation with chemoresistance, we examined the expression profile of genes coding for the NOX2 complex in nine cell lines that are adapted to resist 1 µM of cytarabine (Ara-C) in vitro. Our results showed that the expression of *CYBA* and *NCF4* decreased following Ara-C resistance in these cell lines (Figure S2). Together, these data show that the NOX2 complex is mostly expressed in more mature AML blasts at diagnosis, and is unlikely to contribute to LSC-mediated relapse in AML.

### 3.6. NOX Activity Is Only Detectable after PMA-Induction, Mainly in M4 and M5 AML Subtypes

**Figure 5:**
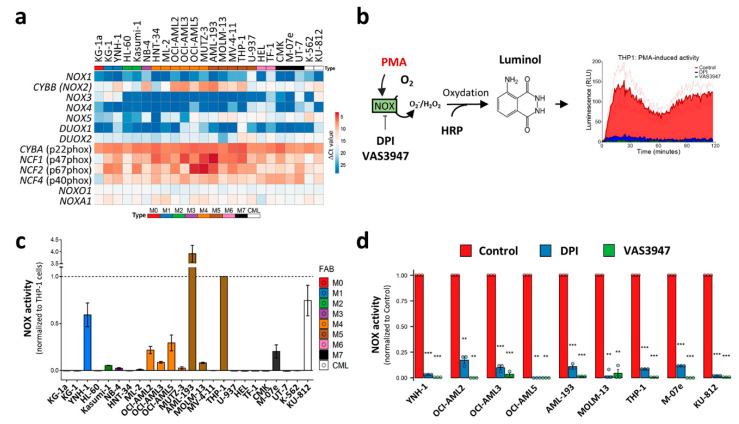
NOX expression and activity in 24 leukaemic cell lines. (**a**) The NOX mRNA expression profile in 22 AML and 2 CML-derived cell lines. The AML cell lines are organized from M0 to M7, according to the FAB classification. The NOX mRNA expression was assessed by RT-qPCR (mean values, N = 3). The heatmap colors reflect the expression level as ΔCt mean values (red: high expression, blue: low expression). (**b**) A schematic representation of the NOX enzymatic activity measurement. PMA induces the assembly of NOX2 complex and stimulates subsequent ROS production, allowing HRP oxidation. In its turn, HRP oxidizes luminol, which emits light that is measured during a 2 h period. (**c**) The PMA-inducible NOX enzymatic activity measurement (N = 3). The NOX activity is reported as area under the curve, normalized to that of THP1 cells. (**d**) The cells were incubated in the presence or absence of DPI (20 µM). The data are shown as the area under the curve (mean ± SEM, N = 3). Student’s t-test was performed for each cell line, comparing the control to each of the DPI- and VAS3947-treated conditions (** *p* < 0.01; *** *p* < 0.001). RLU: relative luminescence unit.

The in silico meta-analysis of the primary AML datasets showed that NOX2 expression is associated with the differentiation stage of AML cells, suggesting that this complex may contribute to ROS production, at least in the M4 and M5 subtypes, with a possible contribution to blast proliferation and differentiation blockage. Previous studies have shown NOX expression and suggested that NOX activity contributes to AML cell proliferation and differentiation [6,26,27]. However, these studies mostly measured ROS production and NOX activity after stimulation or under stress conditions, which can reveal the potential rather than the contribution of NOX to global ROS production in a steady-state. In order to verify whether NOX actively contributes to ROS production in AML cells, we examined *NOX* mRNA levels, together with NOX activity in the absence of stress. In order to avoid contamination by normal cells, such as phagocytes, we chose to work on a broad set of leukemic cell lines that cover all of the FAB stages, rather than on heterogeneous primary cells. In line with the data from primary AML cells, only *CYBB* was highly expressed among the genes coding for catalytical NOX subunits (Figure 5a). As is consistent with the transcriptomic analyses of primary AML, genes coding for NOX2 complex subunits were ubiquitously expressed with higher expression in M4 and M5 cell lines. The Western blot analysis of NOX2 regulatory subunits revealed that their protein profile is correlated with the mRNA levels, especially for NCF1 (p47phox) and NCF2 (p67phox) (Figure S3). The NOX enzymatic activity was then measured in the presence or absence of phorbol myristate acetate (PMA) stimulation (Figure 5b). PMA is an activator of protein kinase C, which can subsequently trigger NOX assembly. Surprisingly, none of the cell lines showed detectable activity in the absence of PMA induction (data not shown); in contrast, PMA induced a detectable enzymatic activity in nine cell lines, mainly those belonging to the M4 (OCI-AML2, OCI-AML3 and OCI-AML5) and M5 (AML-193, MOLM-13 and THP-1) subgroups (Figure 5c). This activity was strongly inhibited by DPI and VAS3947 (82.7% to 99.0%), two widely-used NOX inhibitors (Figure 5d). This indicates that despite the widespread expression of the NOX2 subunits, the NOX2 complex is not readily active in AML cell lines, and requires further stimulation.

### 3.7. CYBB Knockdown (KD) Decreases NOX Activity, but Neither Affects the Proliferation or the Differentiation of AML Cell Lines

The characterization of the AML cell lines led to the identification of two groups: (a) cell lines harboring a low expression of genes coding for the NOX2 complex and lacking NOX2-inducible activity, and (b) cell lines expressing NOX2 subunits along with an inducible NOX2 activity. We further speculated that low NOX activity, below the detection threshold of the enzymatic assay, could still exist and contribute to cell proliferation and differentiation. In order to verify this, we investigated the effect of *CYBB* knockdown (KD) on two representative cell lines.

**Figure 6:**
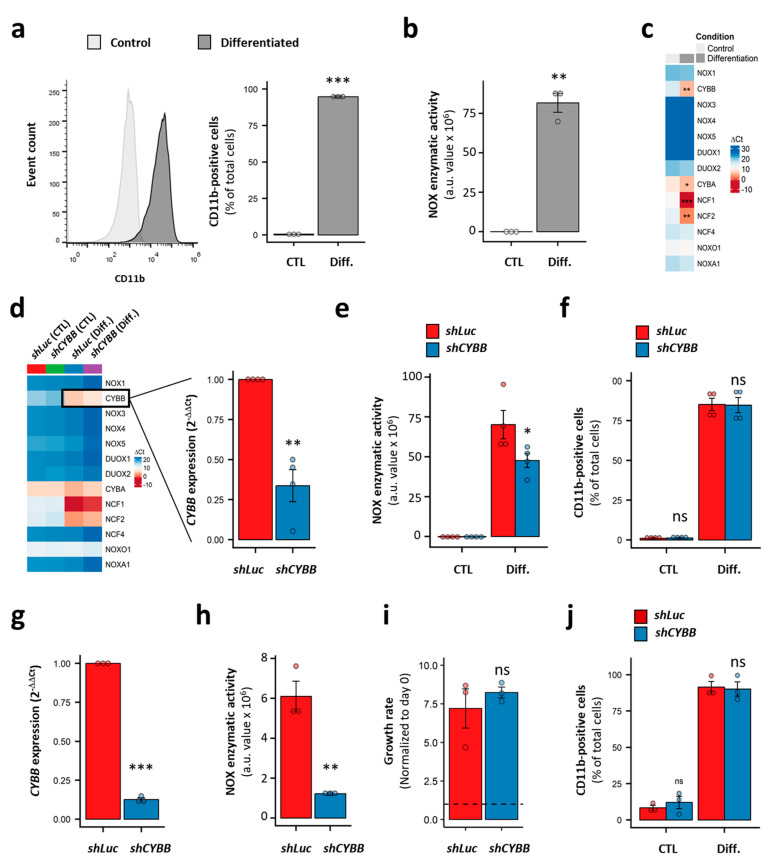
Effect of CYBB knockdown (KD) on the proliferation and differentiation of AML cell lines. (**a**) The expression of CD11b in HL-60 cells with and without differentiation. (**b**) The PMA-induced NOX activity in HL-60 cells, with and without differentiation. (**c**) The NOX expression profile in HL-60 cells, with and without differentiation. (**d**) The effect of CYBB KD on the NOX expression profile in HL-60 cells, with and without differentiation. (**e**) The effect of CYBB KD on PMA-induced NOX activity in HL-60 cells, with and without differentiation. (**f**) The effect of *CYBB* KD on CD11b expression in HL-60 cells, with and without differentiation. The CD11b expression is reported as percentage of positive cells. (**g**–**j**) The effect of *CYBB* KD on *CYBB* expression (**g**), PMA-induced NOX activity (**h**), the proliferation (**i**) and PMA-induced differentiation (**j**) of THP-1 cells. The proliferation of the THP-1 cells was assessed after three days of culture, and the proliferation rate is reported as a ratio to day 0. Student’s t-test, followed by BH adjustment, was used for the pairwise comparisons. (ns: not significant; *: *p* < 0.05; **: *p* < 0.01; ***: *p* < 0.001). CTL: control; Diff.: after differentiation; Luc: luciferase; a.u.: arbitrary unit.

We used HL-60 cells as a model of immature AML cells with no inducible NOX activity (Figure 5c). This cell line is known for its granulocyte- and monocyte/macrophage-like differentiation potential. Indeed, in the presence of DMSO and ATRA, the HL-60 cells fully differentiated, as demonstrated by the expression of the CD11b marker on the cell surface (98.4 ± 0.7% of CD11b^+^ cells; Figure 6a). This was accompanied by the acquisition of PMA-inducible NOX activity (Figure 6b), and a striking increase in the expression of genes coding for the NOX2 complex subunits (Figure 6c). Nonetheless, no activity was detected in the differentiated HL-60 cells in the absence of PMA stimulation (data not shown). Following *CYBB* knockdown, an efficient (69.9 ± 3.2%) decrease in *CYBB* expression was achieved in the differentiated HL-60 cells. Otherwise, it had no effect on genes coding for the other catalytic subunits, indicating an absence of compensation effects (Figure 6d). Although *CYBB* KD was accompanied by a decrease in PMA-induced NOX activity (Figure 6e), it had no effect on the differentiation of HL-60 cells (Figure 6f).

Furthermore, we tested the effect of *CYBB* KD on the more mature THP-1 cell line, which has strong PMA-induced NOX activity. Similarly to the HL-60 cells, the *CYBB* KD in THP-1 cells efficiently decreased the *CYBB* expression and concomitant PMA-induced NOX activity (Figure 6g,h); however, it neither affected cell proliferation, as measured by a trypan blue exclusion assay (Figure 6i) and a resazurin assay (Figure S4), nor their PMA-induced differentiation (Figure 6j). Together, these data indicate that the NOX2 complex does not contribute to the differentiation and proliferation of AML cell lines, and that—even in terminally differentiated cells—NOX2 still requires further stimulation to become active.

## 4. Discussion

Reactive oxygen species play a major role in AML progression. While quiescent LSCs have low levels of ROS, a higher level drives the proliferation of the more mature and metabolically-active blasts. Previous studies have postulated that NOX are central in LSCs, blast proliferation, and differentiation blockage in AML; however, their contribution to ROS production in AML has not been fully addressed.

In this study, we provided a thorough profiling of NOX expression and activity in AML cells using a meta-analysis of public expression datasets, including 839 AML samples of various cytogenetic and molecular subtypes, and the in vitro examination of 24 myeloid leukemia cell lines. Our results demonstrate that genes coding for the NOX2 complex are ubiquitously expressed across blast samples and AML cell lines, and are the most expressed among the 13 NOX genes, in agreement with recent findings [27]. It is worthy of note that although NOX4 expression has been previously described downstream of FLT3-ITD in AML cells [28,54,55], our transcriptional analyses, using three different technologies (microarrays, RNA-seq and qRT-PCR), established that its mRNA expression is lacking from all AML subtypes, including FLT3-ITD. This indicates a possible transcriptional regulation of NOX4 mRNA or high protein stability.

The in-depth survival analysis of the primary AML datasets did not reveal significant prognostic values for CYBB, NCF1, and NCF2 genes, despite a high correlation with three cytogenetic subgroups. In fact, while the t(8;21) and inv(16) subgroups are both associated with good prognosis, they showed opposite expression profiles of NOX2 genes, which could partly explain the lack of consistent global prognosis for NOX. We also noticed high heterogeneity in the NOX profiles within most cytogenetic subgroups, suggesting distinct cellular properties between patients. Our data further show that NOX2 complex subunits are increased in AML cells from the M4 and M5 FAB stages, both at the RNA and protein levels. This is in line with previous observations at the protein level [26,56]. We also found a significant correlation between the mRNA and protein levels for NOX2-regulatory subunits in our cell lines, indicating that the transcriptomic analysis could be reflective of NOX proteins in AML blasts. Interestingly, three NOX2 subunits (CYBB, NCF2 and NCF4) are among a set of 311 genes which we recently identified as being consistently downregulated in the bone marrow (BM) of AML patients from various cytogenetic subgroups, compared to healthy controls [57]. This indicates that bulk AML maintains a lower level of NOX2 compared to more mature cells, confirming the correlation between the expression of the NOX2 complex and the stage of differentiation blockage in AML.

It is well established that ROS can promote the proliferation and apoptosis of cancer cells, which is dependent on its delicate regulation by antioxidant enzymes. While exogenous and metabolic factors can contribute to oxidative stress during AML progression, the role of NOX enzymes at steady states remains elusive. Our transcriptomic meta-analysis revealed that the NOX2 complex is depleted in LSCs and HSCs; however, it is higher in AML blasts, and increases progressively throughout normal granulocytic and monocytic differentiation. Indeed, AML blasts with high ROS levels also have high CYBB expression, whereas AML cells with low ROS levels display lower CYBB expression and higher stemness [8]. Besides this, a correlation between NOX expression and differentiation was established in normal hematopoiesis, in which an increased Nox2 expression and ROS production have been observed in murine granulocyte-monocyte progenitors. However, when we examined 24 AML cell lines, we found that they all lacked detectable NOX activity in the absence of exogenous stimulation. This suggests that NOX2 requires further assembly for its activation in AML cells. In agreement with this, Reddy et al. reported no involvement of NOX in ROS production and oxygen consumption in AML cell lines harboring different tyrosine kinase mutations [25]. Together, these data challenge the previous findings showing a contribution of NOX to ROS production in AML [6]. In fact, Hole et al. reported an endogenous NOX activity in primary AML samples independently of FAB subtypes [6]; however, this study relied on unsorted mononuclear cells, which could be biased by the presence of phagocytes that are characterized by high NOX2 activity. In line with our findings, Hole et al. used PMA stimulation to measure the NOX2 activity in THP-1 cells [6], confirming the lack of endogenous activity in AML cell lines without stimulation. Our data further suggest that increased ROS levels in AML blasts are unlikely to be driven by NOX activity. However, NOX2-generated ROS was recently reported as a key signal for the induction of mitochondrial transfer from stromal cells to AML blasts, as well as in the reduced oxygen consumption [27]; however, these studies did not directly measure the NOX activity in AML cells. Consequently, whether the AML cells in their microenvironment possess an endogenous NOX activity remains an open question.

In our study, the KD of *CYBB* in AML cell lines did not affect the cell proliferation or their differentiation potential. In contrast, Kiffin et al. have shown a decreased differentiation potential of AML cell lines following *CYBB* KD, but—although significant—the difference with normal cells remained quite low. Reddy et al., on the other hand, have shown that CYBB and NOX4 can promote the proliferation and migration of AML cell lines, although independently of their ROS production activity. More recently, Adane et al. demonstrated that *CYBB* KD in primary AML cells results in a slower proliferation, concomitant with an induced differentiation in vitro, and a reduced leukemia burden after xenotransplantation in vivo [27]. Besides this, *Cybb* KO has been shown to reduce the expansion of murine hematopoietic cells transformed with BCR-ABL1 [58], and to deplete LSCs in an AML murine model through the deregulation of genes associated with HSC maintenance [27]. In fact, Adane et al. concluded that NOX2 can regulate the self-renewal of LSCs despite a low expression level [27]. However, the LSC depletion in *Cybb* KO in vivo may originate from chronic inflammation due to the absence of functional phagocytes, rather than a function of endogenous Nox2 itself in LSC homeostasis. Indeed, a murine model of chronic granulomatosis disease lacking functional Nox2 had shown defective HSCs due to chronic inflammation [59]. Thus, the role of NOX in AML malignancy remains controversial; this discrepancy arises, in part, from variabilities between cell lines, murine models, and patient samples, but also from the nature of AML as a heterogenous disease.

Many studies relied on small-molecule inhibitors—such as VAS3947 and DPI—to further elucidate the role of NOX in leukemogenesis, and to examine the therapeutic potential of their inhibition [6,25,27,60,61]. However, most of the inhibitors are known to target multiple enzymatic activities. We recently discovered that VAS3947, which was initially developed as a selective NOX inhibitor [62], induces the apoptosis of AML cells through its aggregation with proteins, independently of NOX inhibition [63]. Likewise, although it is widely used, DPI remains a non-specific pan-NOX inhibitor which can also inhibit other flavoproteins [62]. Therefore, more work needs to be performed before proposing NOX as therapeutic targets in AML.

It is worth noting that some limitations need to be taken into account concerning this work. Although our proteomic and transcriptomic analyses show concordant protein and transcriptional profiles for NOX2 complex genes, it is important to examine the protein localization and surface expression of NOX2 subunits in primary blasts, and to further validate the absence of other NOX proteins at the protein level. Moreover, the functional data reported in this study highlight the effect of *CYBB* KD on AML cell lines under steady-state conditions in vitro. Hence, additional studies are needed to investigate the role of the NOX2 complex under stress conditions, and in the context of primary AML blasts in their microenvironment, or even in animal models in vivo. Furthermore, and importantly, the weak expression of genes coding for the NOX2 complex in LSCs does not necessarily rule out the possibility of weak NOX activity in these cells. Therefore, the investigation of the role of *CYBB* KD in human LSCs is also worth considering in future studies.

## 5. Conclusions

In summary, this work shows that NOX2 is the only NOX complex expressed in AML cells with higher expression in AML committed in the myelomonocytic and monocytic differentiation stages, in contrast to the low expression in LSC and relapsed AML. NOX expression, although it is deregulated in favorable cytogenetic groups, did not correlate with AML prognosis. No endogenous NOX activity was observed in AML cell lines in vitro, although many are inducible upon stimulation. Besides this, *CYBB* KD did not affect the proliferation and differentiation of AML cell lines in vitro. Altogether, our data suggest that NOX genes are differentiation markers with no intrinsic role in AML cells.

## Data Availability

Data is contained within the article or Supplementary Material.

## Supplementary Materials

The following are available online at https://www.mdpi.com/2076-3921/10/3/498/s1, Figure S1: Expression profile of genes coding for NOX2 subunits according to CD34/CD38 status in AML primary cells from the GSE76009 dataset. Figure S2: Transcriptional profile of genes coding for NOX2 subunits following their adaptation to cytarabine (Ara-c) in vitro. Figure S3: protein expression profiles of NOX2 regulatory subunits in AML cell lines and their correlation with RNA levels. Figure S4: THP1 proliferation, as measured by the resazurin assay. Table S1: List of the used cell lines and their culture media. Table S2: Primer sequences for qRT-PCR. Table S3: Summary of the NOX expression in three AML datasets. Table S4: NOX expression according to cytogenetic abnormalities in the three AML expression datasets. Table S5: Univariate cox regression analysis of OS and EFS in three AML datasets. Table S6: Cox regression analysis of NOX in three AML datasets after adjustment for age and cytogenetic abnormalities. Table S7: NOX expression according to molecular abnormalities with cytogenetically normal AML (CN-AML) samples in the three AML expression datasets. Table S8: Univariate cox regression analysis of NOX in the cytogenetically normal (CN-AML) subset of three AML datasets. Table S9: Cox regression analysis of NOX in the cytogenetically normal subset (CN-AML) of three AML datasets after adjustment for age and NPM1/FLT3 status. Table S10: Distribution of FAB subtypes within the cytogenetic groups in AML.

## References

[1] Ghoti H., Amer J., Winder A., Rachmilewitz E., Fibach E. (2007). Oxidative stress in red blood cells, platelets and polymorphonuclear leukocytes from patients with myelodysplastic syndrome. Eur. J. Haematol..

[2] Battisti V., Maders L.D.K., Bagatini M.D., Santos K.F., Spanevello R.M., Maldonado P.A., Brulé A.O., Araújo M.D.C., Schetinger M.R., Morsch V.M. (2008). Measurement of oxidative stress and antioxidant status in acute lymphoblastic leukemia patients. Clin. Biochem..

[3] Hole P.S., Darley R.L., Tonks A. (2011). Do reactive oxygen species play a role in myeloid leukemias?. Blood.

[4] Holmström K.M., Finkel T. (2014). Cellular mechanisms and physiological consequences of redox-dependent signalling. Nat. Rev. Mol. Cell Biol..

[5] Ray P.D., Huang B.W., Tsuji Y. (2012). Reactive oxygen species (ROS) homeostasis and redox regulation in cellular signaling. Cell. Signal..

[6] Hole P.S., Zabkiewicz J., Munje C., Newton Z., Pearn L., White P., Marquez N., Hills R.K., Burnett A.K., Tonks A. (2013). Overproduction of NOX-derived ROS in AML promotes proliferation and is associated with defective oxidative stress signaling. Blood.

[7] Herault O., Hope K.J., Deneault E., Mayotte N., Chagraoui J., Wilhelm B.T., Cellot S., Sauvageau M., Andrade-Navarro M.A., Hébert J. (2012). A role for GPx3 in activity of normal and leukemia stem cells. J. Exp. Med..

[8] Lagadinou E.D., Sach A., Callahan K., Rossi R.M., Neering S.J., Minhajuddin M., Ashton J.M., Pei S., Grose V., O’Dwyer K.M. (2013). BCL-2 inhibition targets oxidative phosphorylation and selectively eradicates quiescent human leukemia stem cells. Cell Stem Cell.

[9] Valko M., Leibfritz D., Moncol J., Cronin M.T.D., Mazur M., Telser J. (2007). Free radicals and antioxidants in normal physiological functions and human disease. Int. J. Biochem. Cell Biol..

[10] Lambeth J.D., Neish A.S. (2014). Nox enzymes and new thinking on reactive oxygen: A double-edged sword revisited. Annu. Rev. Pathol. Mech. Dis..

[11] Nisimoto Y., Diebold B.A., Constentino-Gomes D., Lambeth J.D. (2014). Nox4: A hydrogen peroxide-generating oxygen sensor. Biochemistry.

[12] Takac I., Schröder K., Zhang L., Lardy B., Anilkumar N., Lambeth J.D., Shah A.M., Morel F., Brandes R.P. (2011). The E-loop is involved in hydrogen peroxide formation by the NADPH oxidase Nox4. J. Biol. Chem..

[13] Brar S.S., Kennedy T.P., Sturrock A.B., Huecksteadt T.P., Quinn M.T., Whorton R.A., Hoidal J.R. (2002). An NAD(P)H oxidase regulates growth and transcription in melanoma cells. Am. J. Physiol. Cell Physiol..

[14] Raad H., Serrano-Sanchez M., Harfouche G., Mahfouf W., Bortolotto D., Bergeron V., Kasraian Z., Dousset L., Hosseini M., Taieb A. (2017). NADPH oxidase-1 plays a key role in keratinocyte responses to UV radiation and UVB-induced skin carcinogenesis. J. Investig. Dermatol..

[15] Rezvani H.R., Rossignol R., Ali N., Benard G., Tang X., Yang H.S., Jouary T., De Verneuil H., Taïeb A., Kim A.L. (2011). XPC silencing in normal human keratinocytes triggers metabolic alterations through NOX-1 activation-mediated reactive oxygen species. Biochim. Biophys. Acta Bioenerg..

[16] Brar S.S., Corbin Z., Kennedy T.P., Hemendinger R., Thornton L., Bommarius B., Arnold R.S., Whorton A.R., Sturrock A.B., Huecksteadt T.P. (2003). NOX5 NAD(P)H oxidase regulates growth and apoptosis in DU 145 prostate cancer cells. Am. J. Physiol. Cell Physiol..

[17] Fukuyama M., Rokutan K., Sano T., Miyake H., Shimada M., Tashiro S. (2005). Overexpression of a novel superoxide-producing enzyme, NADPH oxidase 1, in adenoma and well differentiated adenocarcinoma of the human colon. Cancer Lett..

[18] Lu W., Hu Y., Chen G., Chen Z., Zhang H., Wang F., Feng L., Pelicano H., Wang H., Keating M.J. (2012). Novel role of NOX in supporting aerobic glycolysis in cancer cells with mitochondrial dysfunction and as a potential target for cancer therapy. PLoS Biol..

[19] Lambeth J.D. (2007). Nox enzymes, ROS, and chronic disease: An example of antagonistic pleiotropy. Free Radic. Biol. Med..

[20] Blanchetot C., Boonstra J. (2008). The ROS-NOX connection in cancer and angiogenesis. Crit. Rev. Eukaryot. Gene Expr..

[21] Chen C., Li L., Zhou H.J., Min W. (2017). The role of NOX4 and TRX2 in angiogenesis and their potential cross-talk. Antioxidants.

[22] Bonner M.Y., Arbiser J.L. (2012). Targeting NADPH oxidases for the treatment of cancer and inflammation. Cell. Mol. Life Sci..

[23] Weyemi U.E., Redon C.R., Parekh P., Dupuy C.M., Bonner W. (2013). NADPH oxidases NOXs and DUOXs as putative targets for cancer therapy. Anticancer Agents Med. Chem..

[24] Jayavelu A.K., Moloney J.N., Böhmer F.D., Cotter T.G. (2016). NOX-driven ROS formation in cell transformation of FLT3-ITD-positive AML. Exp. Hematol..

[25] Reddy M.M., Fernandes M.S., Salgia R., Levine R.L., Griffin J.D., Sattler M. (2011). NADPH oxidases regulate cell growth and migration in myeloid cells transformed by oncogenic tyrosine kinases. Leukemia.

[26] Kiffin R., Wiktorin H.G., Nilsson M.S., Aurelius J., Aydin E., Lenox B., Nilsson J.A., Ståhlberg A., Thorén F.B., Hellstrand K. (2018). Anti-leukemic properties of histamine in monocytic leukemia: The role of NOX2. Front. Oncol..

[27] Adane B., Ye H., Khan N., Pei S., Minhajuddin M., Stevens B.M., Jones C.L., D’Alessandro A., Reisz J.A., Zaberezhnyy V. (2019). The Hematopoietic Oxidase NOX2 Regulates Self-Renewal of Leukemic Stem Cells. Cell Rep..

[28] Moloney J.N., Stanicka J., Cotter T.G. (2017). Subcellular localization of the FLT3-ITD oncogene plays a significant role in the production of NOX- and p22phox-derived reactive oxygen species in acute myeloid leukemia. Leuk. Res..

[29] Gautier L., Cope L., Bolstad B.M., Irizarry R.A. (2004). Affy—Analysis of Affymetrix GeneChip data at the probe level. Bioinformatics.

[30] R Core Team (2018). R: A Language and Environment for Statistical Computing.

[31] Novershtern N., Subramanian A., Lawton L.N., Mak R.H., Haining W.N., McConkey M.E., Habib N., Yosef N., Chang C.Y., Shay T. (2011). Densely interconnected transcriptional circuits control cell states in human hematopoiesis. Cell.

[32] Hackl H., Steinleitner K., Lind K., Hofer S., Tosic N., Pavlovic S., Suvajdzic N., Sill H., Wieser R. (2015). A gene expression profile associated with relapse of cytogenetically normal acute myeloid leukemia is enriched for leukemia stem cell genes. Leuk. Lymphoma.

[33] Ng S.W.K., Mitchell A., Kennedy J.A., Chen W.C., McLeod J., Ibrahimova N., Arruda A., Popescu A., Gupta V., Schimmer A.D. (2016). A 17-gene stemness score for rapid determination of risk in acute leukaemia. Nature.

[34] Perez-Riverol Y., Csordas A., Bai J., Bernal-Llinares M., Hewapathirana S., Kundu D.J., Inuganti A., Griss J., Mayer G., Eisenacher M. (2019). The PRIDE database and related tools and resources in 2019: Improving support for quantification data. Nucleic Acids Res..

[35] Hernandez-Valladares M., Aasebø E., Berven F., Selheim F., Bruserud Ø. (2020). Biological characteristics of aging in human acute myeloid leukemia cells: The possible importance of aldehyde dehydrogenase, the cytoskeleton and altered transcriptional regulation. Aging.

[36] Zhang X., Smits A.H., Van Tilburg G.B.A., Ovaa H., Huber W., Vermeulen M. (2018). Proteome-wide identification of ubiquitin interactions using UbIA-MS. Nat. Protoc..

[37] Wickham H. (2016). ggplot2: Elegant Graphics for Data Analysis.

[38] Gu Z., Eils R., Schlesner M. (2016). Complex heatmaps reveal patterns and correlations in multidimensional genomic data. Bioinformatics.

[39] Wei T., Simko V. (2016). Visualization of a Correlation Matrix: Package “corrplot”. Statistician.

[40] Metsalu T., Vilo J. (2015). ClustVis: A web tool for visualizing clustering of multivariate data using Principal Component Analysis and heatmap. Nucleic Acids Res..

[41] Cox D.R. (1972). Regression models and life-tables. J. R. Stat. Soc. Ser. B.

[42] Schoenfeld D. (1982). Partial residuals for the proportional hazards regression model. Biometrika.

[43] Therneau T.M., Grambsch P.M. (2000). Modeling Survival Data: Extending the Cox Model.

[44] Brachet-Botineau M., Deynoux M., Vallet N., Polomski M., Juen L., Hérault O., Mazurier F., Viaud-Massuard M.-C., Prié G., Gouilleux F. (2019). A novel inhibitor of stat5 signaling overcomes chemotherapy resistance in myeloid leukemia cells. Cancers.

[45] Kouzi F., Zibara K., Bourgeais J., Picou F., Gallay N., Brossaud J., Dakik H., Roux B., Hamard S., Le Nail L.-R. (2020). Disruption of gap junctions attenuates acute myeloid leukemia chemoresistance induced by bone marrow mesenchymal stromal cells. Oncogene.

[46] Rezvani H.R., Kim A.L., Rossignol R., Ali N., Daly M., Mahfouf W., Bellance N., Taieb A., de Verneuil H., Mazurier F. (2011). XPC silencing in normal human keratinocytes triggers metabolic alterations that drive the formation of squamous cell carcinomas. J. Clin. Invest..

[47] Kang C., Kim C.Y., Kim H.S., Park S.P., Chung H.M. (2018). The bromodomain inhibitor JQ1 enhances the responses to all-trans retinoic acid in HL-60 and MV4-11 leukemia cells. Int. J. Stem Cells.

[48] Verhaak R.G.W., Wouters B.J., Erpelinck C.A.J., Abbas S., Beverloo H.B., Lugthart S., Löwenberg B., Delwel H., Valk P. (2009). Prediction of molecular subtypes in acute myeloid leukemia based on gene expression profiling. Haematologica.

[49] Tomasson M.H., Xiang Z., Walgren R., Zhao Y., Kasai Y., Miner T., Ries R.E., Lubman O., Fremont D.H., McLellan M.D. (2008). Somatic mutations and germline sequence variants in the expressed tyrosine kinase genes of patients with de novo acute myeloid leukemia. Blood.

[50] Ley T.J., Miller C., Ding L., Raphael B.J., Mungall A.J., Robertson G., Hoadley K., Triche T.J., Laird P.W., Baty J.D. (2013). Genomic and epigenomic landscapes of adult de novo acute myeloid leukemia. N. Engl. J. Med..

[51] Bennett J.M., Catovsky D., Daniel M.-T., Flandrin G., Galton D.A.G., Gralnick H.R., Sultan C. (1976). Proposals for the classification of the acute leukaemias French-American-British (FAB) co-operative group. Br. J. Haematol..

[52] Bennett J.M., Catovsky D., Daniel M.T., Flandrin G., Galton D.A., Gralnick H.R., Sultan C. (1985). Criteria for the diagnosis of acute leukemia of megakaryocyte lineage (M7). A report of the French-American-British Cooperative Group. Ann. Intern. Med..

[53] Sangle N.A., Perkins S.L. (2011). Core-binding factor acute myeloid leukemia. Arch. Pathol. Lab. Med..

[54] Woolley J.F., Naughton R., Stanicka J., Gough D.R., Bhatt L., Dickinson B.C., Chang C.J., Cotter T.G. (2012). H 2 O 2 Production Downstream of FLT3 Is Mediated by p22phox in the Endoplasmic Reticulum and Is Required for STAT5 Signalling. PLoS ONE.

[55] Jayavelu A.K., Müller J.P., Bauer R., Böhmer S.A., Lässig J., Cerny-Reiterer S., Sperr W.R., Valent P., Maurer A.B., Moriggl R. (2016). NOX4-driven ROS formation mediates PTP inactivation and cell transformation in FLT3ITD-positive AML cells. Leukemia.

[56] Aurelius J., Hallner A., Werlenius O., Riise R., Mollgard L., Brune M., Hansson M., Martner A., Thoren F.B., Hellstrand K. (2017). NOX2-dependent immunosuppression in chronic myelomonocytic leukemia. J. Leukoc. Biol..

[57] Nehme A., Dakik H., Picou F., Cheok M., Preudhomme C., Dombret H., Lambert J., Gyan E., Pigneux A., Récher C. (2020). Horizontal meta-analysis identifies common deregulated genes across AML subgroups providing a robust prognostic signature. Blood Adv..

[58] Grauers Wiktorin H., Nilsson T., Aydin E., Hellstrand K., Palmqvist L., Martner A. (2018). Role of NOX2 for leukaemic expansion in a murine model of BCR-ABL1+ leukaemia. Br. J. Haematol..

[59] Weisser M., Demel U.M., Stein S., Chen-Wichmann L., Touzot F., Santilli G., Sujer S., Brendel C., Siler U., Cavazzana M. (2016). Hyperinflammation in patients with chronic granulomatous disease leads to impairment of hematopoietic stem cell functions. J. Allergy Clin. Immunol..

[60] Sanchez-Sanchez B., Gutierrez-Herrero S., Lopez-Ruano G., Prieto-Bermejo R., Romo-Gonzalez M., Llanillo M., Pandiella A., Guerrero C., Miguel J.F.S., Sánchez-Guijo F.M. (2014). NADPH oxidases as therapeutic targets in chronic myelogenous leukemia. Clin. Cancer Res..

[61] Farge T., Saland E., de Toni F., Aroua N., Hosseini M., Perry R., Bosc C., Sugita M., Stuani L., Fraisse M. (2017). Chemotherapy-resistant human acute myeloid leukemia cells are not enriched for leukemic stem cells but require oxidative metabolism. Cancer Discov..

[62] Wind S., Beuerlein K., Eucker T., Müller H., Scheurer P., Armitage M.E., Ho H., Schmidt H.H.H.W., Wingler K. (2010). Comparative pharmacology of chemically distinct NADPH oxidase inhibitors. Br. J. Pharmacol..

[63] El Dor M., Dakik H., Polomski M., Haudebourg E., Brachet M., Gouilleux F., Prié G., Zibara K., Mazurier F. (2020). Vas3947 induces upr-mediated apoptosis through cysteine thiol alkylation in aml cell lines. Int. J. Mol. Sci..

