# Supplementary material for: Characterization of NADPH Oxidase Expression and Activity in Acute Myeloid Leukemia Cell Lines: A Correlation with the Differentiation Status
Source: Antioxidants (Basel). 2021 Mar 23;10(3):498. doi: 10.3390/antiox10030498 (PMC8004739; doi:10.3390/antiox10030498)
Supplement: Supplementary file 1 [file antioxidants-10-00498-s001.pdf]

antioxidants

Supplementary Materials: Characterization of NADPH Oxidase Expression and Activity in Acute Myeloid Leukemia Cell Lines: A Correlation with the Differentiation Status

Hassan Dakik [1,2,†,*], Maya El Dor [1,2,†], Joan Leclerc [1], Farah Kouzi [1,2], Ali Nehme [1,2], Margaux Deynoux [1], Christelle Debeissat [1], Georges Khamis [1], Elfi Ducrocq [1], Aida Ibrik [2,6], Marie-José Stasia [3,4], Houssam Raad [5], Hamid Reza Rezvani [5], Fabrice Gouilleux [1,7], Kazem Zibara [2,6,*], Olivier Herault [1,7,8] and Frédéric Mazurier [1,7,*]

[1] University of Tours EA 7501, CNRS ERL 7001, LNOx Team, F-37032 Tours, France; hassan.dakik@mail.mcgill.ca (H.D.); maya.el-dor@etu.univ-tours.fr (M.E.D.); leclerc_joan@yahoo.fr (J.L.); farah.kouzi@etu.univ-tours.fr (F.K.); ali.nehme2@mcgill.ca (A.N.); margaux.deynoux@etu.univ-tours.fr (M.D.); christelle.debeissat@u-bordeaux.fr (C.D.); georges.khamis@univ-tours.fr (G.K.); elfi.ducrocq@univ-tours.fr (E.D.); fabrice.gouilleux@univ-tours.fr (F.G.); olivier.herault@univ-tours.fr (O.H.)

[2] PRASE, Lebanese University, 6573/14 Beirut, Lebanon; aida.ibrik@ul.edu.lb

[3] University Grenoble Alpes, CEA, CNRS, IBS, F-38044 Grenoble, France; MJStasia@chu-grenoble.fr

[4] CDiReC, Pôle Biologie, CHU de Grenoble, F-38043 Grenoble, France

[5] University of Bordeaux, INSERM U1035, F-33000 Bordeaux, France; houssam.raad@ul.edu.lb(H.R.); hamid-reza.rezvani@u-bordeaux.fr (H.R.R.)

[6] Biology Department, Faculty of Sciences - I, Lebanese University, 90656 Beirut, Lebanon

[7] CNRS GDR 3697 MicroNiT, F-37032 Tours, France

[8] CHRU de Tours, Service d'Hématologie Biologique, F-37000 Tours, France

* Correspondence: hassan.dakik@mail.mcgill.ca (H.D.); kzibara@ul.edu.lb (K.Z.); frederic.mazurier@inserm.fr (F.M.)

† These authors contributed equally to the work.

Citation: Dakik, H.; El Dor, M.; Leclerc, J.; Kouzi, F.; Nehme, A.; Deynoux, M.; Debeissat, C.; Khamis, G.; Ducrocq, E.; Ibrik, A.; et al. Characterization of NADPH Oxidase Expression and Activity in Acute Myeloid Leukemia Cell Lines: A Correlation with the Differentiation Status. *Antioxidants* 2021, *10*, 498. https://doi.org/10.3390/antiox10030498

Academic Editor: Antonella Pantaleo, Christian Secchi and Marco Orecchioni

Received: 7 February 2021
Accepted: 11 March 2021
Published: 23 March 2021

Publisher's Note: MDPI stays neutral with regard to jurisdictional claims in published maps and institutional affiliations.

Copyright: © 2021 by the authors. Submitted for possible open access publication under the terms and conditions of the Creative Commons Attribution (CC BY) license (http://creativecommons.org/licenses/by/4.0/).

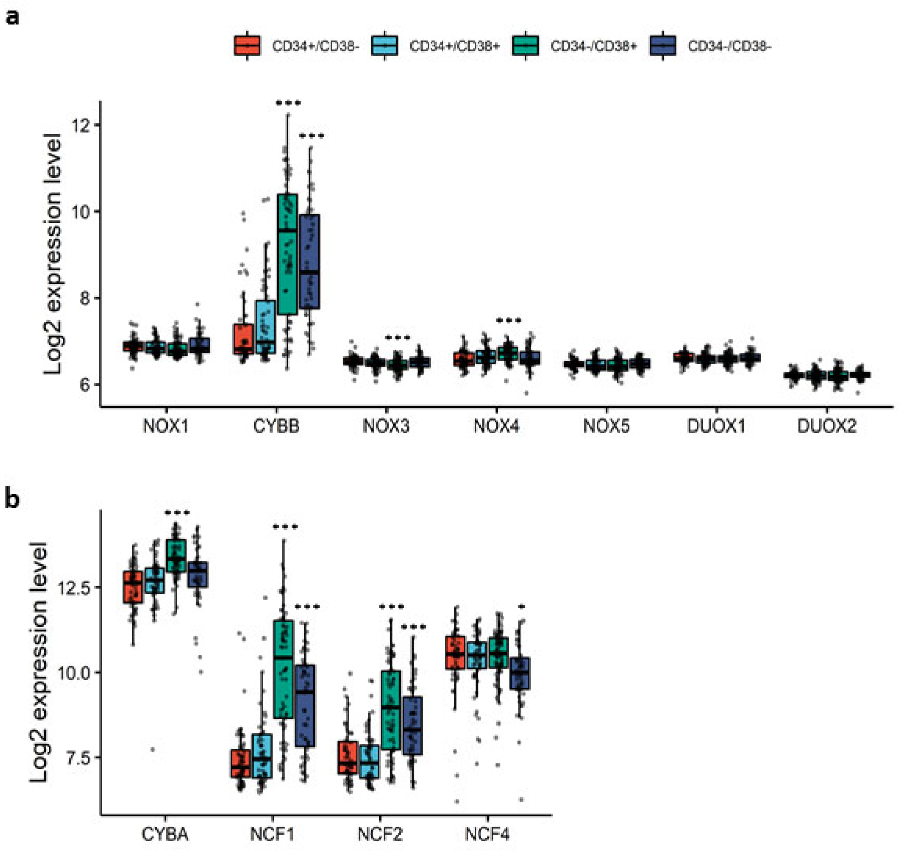

Figure S1. Expression profile of genes coding for NOX2 subunits according to CD34/CD38 status in AML primary cells from GSE76009 dataset. a) Expression profile of genes coding for NOX catalytical subunits. b) Expression profile of genes coding for NOX2 regulatory subunits. Student's t-test followed by BH-adjustment was used for pairwise comparisons between $CD34^{+}CD38^{-}$ population and the other populations. (*: P <.05; ***: P <.001).

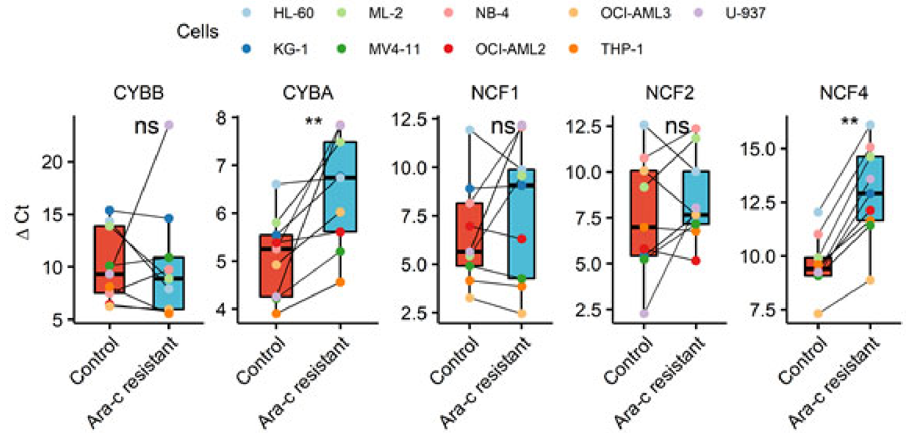

Figure S2. Transcriptional profile of genes coding for NOX2 subunits following adaptation to cytarabine (Ara-c) *in vitro*. AML cell lines were exposed to increasing doses of Ara-c until resistance to a dose of 1 μM was reached. Wilcoxon's signed-rank test was used for pairwise comparisons between Ara-c resistant cells and controls. (**: $p < 0.01$).

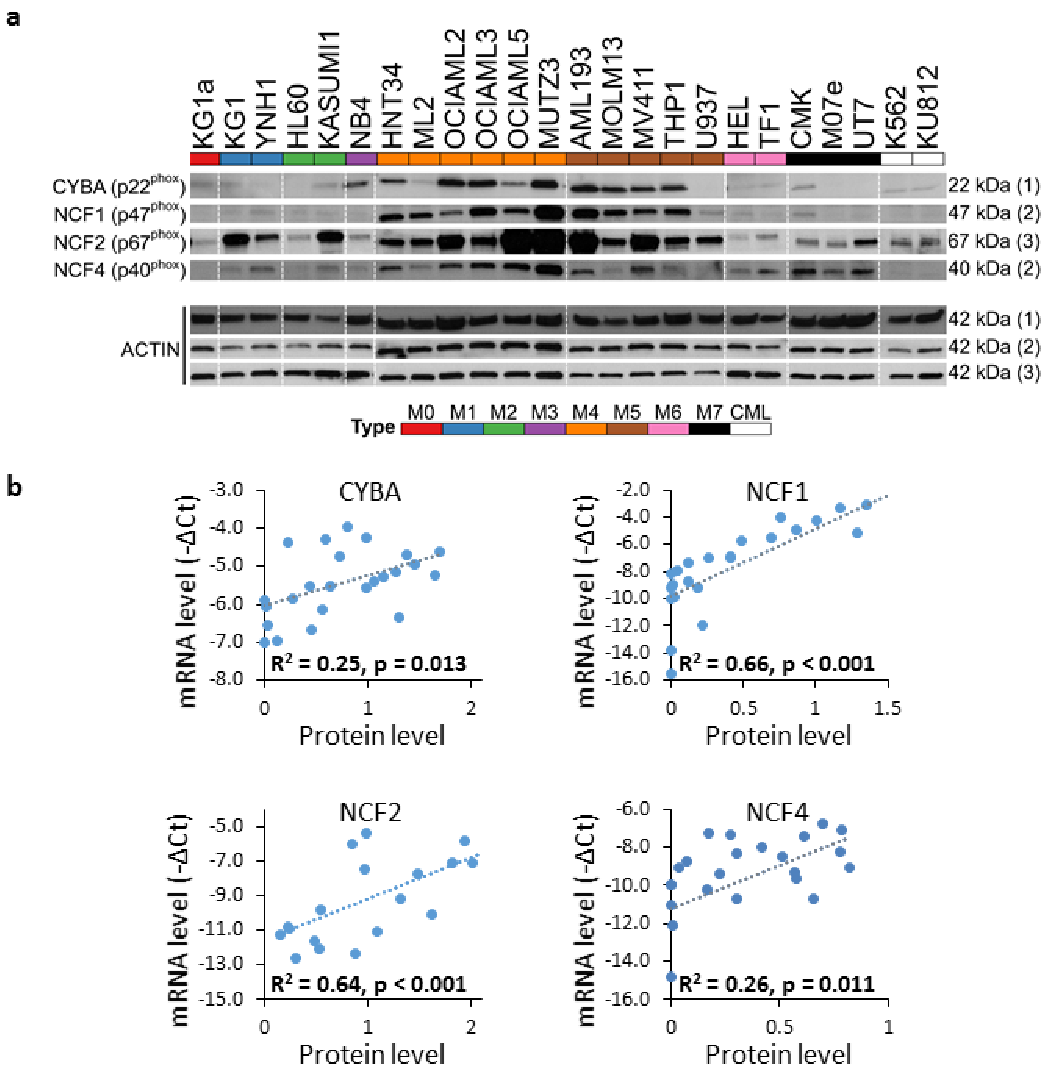

Figure S3. protein expression profiles of NOX2 regulatory subunits in AML cell lines and their correlation with RNA levels. a) Western blot analysis of CYBA (p22phox), NCF1 (p47phox), NCF2 (p67phox) and NCF4 (p40phox) in 24 myeloid leukemia cell lines. Expression profile of genes coding for NOX catalytical subunits. The numbers on the right associate regulatory protein with their Actin control. **b)** Pearson correlation between protein and mRNA levels of NOX2 regulatory subunits in the 24 cell lines. Protein expression levels were calculated as ratio to Actin.

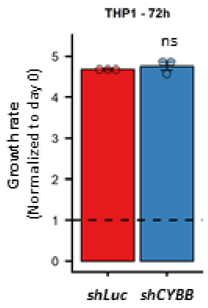

Figure S4. THP1 proliferation as measured by the resazurin assay. The proliferation of THP-1 cells was assessed after three days of culture, and proliferation rate is reported as a ratio to day 0. Student's t-test was used for pairwise comparison.

Table S1. List of used cell lines and their culture media.

Type	Cell line	Subtype	Culture medium		
			Media*	FBS (%)**	Cytokines
AML	KG-1a	M0	RPMI	10	
	KG-1	M1	RPMI	10	
	YNH-1	M1	RPMI	20	GM-CSF (10 ng/ml)
	HL-60	M2	RPMI	10	
	Kasumi-1	M2	RPMI	20	
	NB-4	M3	RPMI	10	
	ML-2	M4	RPMI	10	
	OCI-AML3	M4	RPMI	20	
	OCI-AML2	M4	α-MEM	20	
	OCI-AML5	M4	α-MEM	20	GM-CSF (10 ng/ml)
	MUTZ-3	M4	α-MEM	20	GM-CSF (10 ng/ml)
	HNT-34	M4	RPMI	10	
	MV-4-11	M5	RPMI	10	
	MOLM-13	M5	RPMI	10	
	THP-1	M5	RPMI	10	
	AML-193	M5	RPMI	10	GM-CSF (10 ng/ml)
	U-937	M5	RPMI	10	
	HEL	M6	RPMI	10	
	TF-1	M6	RPMI	20	GM-CSF (5 ng/ml)
	UT-7	M7	α-MEM	20	GM-CSF (5 ng/ml)
	CMK	M7	RPMI	10	
	M-07e	M7	RPMI	10	GM-CSF (10 ng/ml)
CML	K-562		RPMI	10	
	KU-812		RPMI	20	

*Media were supplemented with 2 mM L-glutamine (Life Technologies, Villebon-sur-Yvette, France), and 100 units/mL penicillin G, and 100 µg/mL streptomycin (Boehringer-Mannheim, Mannheim, Germany).

**FBS (Life Technologies) was heat-decomplemented prior to use.

Abbreviations: AML: Acute myeloid Leukemia. CML: Chronic Myeloid Leukemia. FBS: Fetal bovine serum.

Table S2. Primers sequences for qRT-PCR.

Gene symbol	Primer (Forward)	Primer (Reverse)	Probe
NOX1	AAGGATCCTCCGGTTTTACC	TTTGGATGGGTGCATAACAA	CTGCTGGG
CYBB (NOX2)	GAAGAAAGGCAAACACAACACA	CCCCAGCCAAACCAGAAT	TGGCAGAG
NOX3	CGAGAGCTACCTCAACCCTGT	TGACGCCTGCTATTGTCCTT	TGGGGAAG
NOX4	GCTGACGTTGCATGTTTCAG	CGGGAGGGTGGGTATCTAA	GGCTGCTG
NOX5	CCCTTCACCATCAGCAGTG	TGTTTGTCCACTGGCCTTG	CTGGCTGC
DUOX1	CACCTCCTGGAGACCTTTTTC	GTCGGCCTGGTTGATGTC	TCTCCCAG
DUOX2	TGCATATTCCCCAACGTCTT	GGTCTGGAAGAACCACCAATAG	TGGGGAAG
CYBA (*p22*^{phox})	GAGCGGCATCTACCTACTGG	TGATGGTGCCTCCGATCT	GCAGTGGA
NCF1 (*p47*^{phox})	CCTGCTGGGCTTTGAGAA	GACAGGTCCTGCCATTTCAC	CCAGCCAG
NCF2 (*p67*^{phox})	CTCTGGGTTTGCCCCTCT	TCTCTGGGGTTTTCGGTCT	CAGGCAGC
NCF4 (*p40*^{phox})	TTTGCAGAGCAAGCTGGAG	TCCTGTTTCACACCCACGTA	TGCCCTGG
NOXO1	CAGGAGAGCCTGGACGTG	CTGCCGGTCTTCGTTCTC	GGTGGCTG
NOXA1	GTCACGGCTTGGTCAAATG	GCCAGGCTGTGCTTCAAC	CAGCAGGT
ACTB	ATTGGCAATGAGCGGTTC	CGTGGATGCCACAGGACT	GCTGGAAG
YWHAZ	GCAATTACTGAGAGACAACTTGACA	TGGAAGGCCGGTTAATTTT	TTCTCCTG
RPL13A	CAAGCGGATGAACACCAAC	TGTGGGGCAGCATACCTC	CCAGCCGC

antioxidants

MDPI

Table S3. Summary of NOX expression in 3 AML datasets.

Gene	GSE6891 (N = 443)					GSE10358 (N = 223)					TCGA (N = 173)				
	Log2 expression	Min	Max	Median	SD	Log2 expression	Min	Max	Median	SD	Log2 expression	Min	Max	Median	SD
NOX1	2.24	2.23	4.01	2.23	0.10	4.82	4.09	7.07	4.77	0.41	0.76	0.00	3.03	0.77	0.80
CYBB	**9.13**	2.39	13.82	10.05	3.08	**7.28**	3.95	11.06	7.43	1.94	**11.57**	5.63	16.02	12.22	2.33
NOX3	2.26	2.23	9.54	2.23	0.40	2.70	2.30	4.86	2.66	0.24	0.14	0.00	4.80	0.00	0.55
NOX4	2.23	2.23	2.88	2.23	0.03	2.77	2.21	4.43	2.72	0.27	0.04	0.00	2.52	0.00	0.25
NOX5	2.65	2.23	6.56	2.60	0.29	5.83	5.24	8.04	5.74	0.42	1.21	0.00	7.12	0.77	1.64
DUOX1	2.95	2.23	4.03	2.92	0.24	3.84	3.08	5.24	3.82	0.29	5.18	2.81	7.73	5.20	0.83
DUOX2	2.23	2.23	2.27	2.23	0.00	3.05	2.32	4.47	3.02	0.31	1.54	0.00	6.20	1.32	1.38
CYBA	**12.29**	8.90	14.24	12.45	0.89	**10.63**	7.47	12.11	10.79	0.74	**12.22**	10.12	14.78	12.26	0.73
NCF1	**8.90**	4.21	14.01	8.57	2.09	**6.14**	3.44	10.34	5.78	1.75	**8.55**	2.62	13.33	8.59	2.41
NCF2	**8.72**	2.23	13.68	9.71	3.54	**7.01**	2.71	10.54	7.05	2.12	**10.09**	4.12	13.59	10.51	2.07
NCF4	**10.32**	3.76	11.97	10.58	1.06	**7.92**	4.27	9.84	8.13	0.95	**10.75**	6.79	12.79	10.83	0.81
NOXA1	6.06	4.26	7.66	5.99	0.24	5.64	3.90	8.22	5.66	0.52	4.62	0.00	8.88	4.76	1.78
NOXO1	2.23	2.23	2.54	2.23	0.01	3.75	2.74	5.67	3.74	0.32	2.06	0.00	4.21	2.04	0.85

Table S4. NOX expression according to cytogenetic abnormalities in the 3 AML expression datasets.

		GSE6891 (N=404)				GSE10358 (N=220)				TCGA (N=170)			
Gene	Comparison	FC	p-value	q-value		FC	p-value	q-value		FC	p-value	q-value	
	**Normal karyotype vs the rest**	0	0.6174	0.6174		0	0.9393	0.9393		-0.15	0.2334	0.434	
	**t(8;21) vs the rest**	-0.02	0.0068	0.0295	*	0.06	0.5353	0.6202		0.54	0.1223	0.199	
	**t(15;17) vs the rest**	0.02	0.5284	0.5724		0.17	0.0383	0.0996		-0.08	0.6671	0.788	
NOX1	**inv(16) vs the rest**	-0.01	0.4916	0.5326		-0.01	0.94	0.94		0.32	0.2948	0.348	
	**Intermediate Risk Cytogenetics vs the rest**	-0.01	0.4377	0.4742		-0.15	0.0172	0.0559		-0.02	0.9442	0.944	
	**Complex Cytogenetics vs the rest**	0.13	0.3729	0.606		-0.05	0.782	0.8156		-0.11	0.5167	0.84	
	**Poor Risk Cytogenetics vs the rest**	-0.01	0.2193	0.4752		0.01	0.9517	0.989		0.18	0.3532	0.647	
	**Normal karyotype vs the rest**	0.39	0.2047	0.4435		0.05	0.8607	0.9324		0.14	0.6982	0.756	
	**t(8;21) vs the rest**	**-2.62**	<0.001	<0.001	***	**-2.18**	<0.001	<0.001	***	**-4.18**	<0.001	<0.001	***
	**t(15;17) vs the rest**	0.72	0.1514	0.3286		-0.4	0.2212	0.2876		0.53	0.1347	0.292	
CYBB	**inv(16) vs the rest**	**2.68**	<0.001	<0.001	***	**1.58**	<0.001	0.0022	**	**2.12**	<0.001	<0.001	***
	**Intermediate Risk Cytogenetics vs the rest**	-0.9	0.0303	0.1465		0.32	0.3438	0.4063		0.65	0.2161	0.702	
	**Complex Cytogenetics vs the rest**	-0.02	0.979	0.9993		-0.31	0.4778	0.7886		-0.29	0.5156	0.84	
	**Poor Risk Cytogenetics vs the rest**	0.05	0.9204	0.9204		0.29	0.5366	0.9675		-0.71	0.208	0.647	
	**Normal karyotype vs the rest**					-0.04	0.2257	0.392		-0.14	0.0833	0.371	
	**t(8;21) vs the rest**					0.11	0.1545	0.3348		0.76	0.2389	0.345	
	**t(15;17) vs the rest**					0.08	0.0934	0.2024		-0.16	0.0008	0.004	**
NOX3	**inv(16) vs the rest**		NA			-0.06	0.1327	0.327		-0.15	0.0009	0.003	**
	**Intermediate Risk Cytogenetics vs the rest**					-0.07	0.0432	0.0873		0.02	0.8025	0.944	
	**Complex Cytogenetics vs the rest**					-0.1	0.048	0.312		0	0.9683	0.968	
	**Poor Risk Cytogenetics vs the rest**					0.22	0.1787	0.7744		0.23	0.3779	0.647	
	**Normal karyotype vs the rest**					-0.05	0.1723	0.392					
	**t(8;21) vs the rest**					0.04	0.5725	0.6202					
	**t(15;17) vs the rest**					0.1	0.1334	0.2168					
NOX4	**inv(16) vs the rest**		NA			0	0.9269	0.94			NA		
	**Intermediate Risk Cytogenetics vs the rest**					-0.09	0.0107	0.0464	*				
	**Complex Cytogenetics vs the rest**					0.01	0.8156	0.8156					
	**Poor Risk Cytogenetics vs the rest**					0.14	0.0587	0.7631					
	**Normal karyotype vs the rest**	0.02	0.5783	0.6174		0.07	0.2362	0.392		0.42	0.1044	0.371	
	**t(8;21) vs the rest**	0.02	0.6411	0.6945		0.1	0.3852	0.5564		**-1.28**	<0.001	<0.001	***
NOX5	**t(15;17) vs the rest**	-0.05	0.0013	0.0056	**	0.12	0.1209	0.2168		0.7	0.2189	0.407	
	**inv(16) vs the rest**	0.02	0.6415	0.6415		-0.05	0.6339	0.94		-0.38	0.4971	0.497	

	Intermediate Risk Cytogenetics vs the rest	0.02	0.7012	0.7012		-0.15	0.0074	0.0464	*	-0.37	0.3166	0.823	
	Complex Cytogenetics vs the rest	-0.04	0.0427	0.2776		-0.13	0.4536	0.7886		-0.34	0.2666	0.84	
	Poor Risk Cytogenetics vs the rest	-0.06	<0.001	0.0052	**	-0.04	0.7442	0.9675		-0.15	0.6457	0.763	
DUOX1	Normal karyotype vs the rest	-0.01	0.6038	0.6174		-0.06	0.1414	0.392		-0.18	0.1749	0.434	
	t(8;21) vs the rest	0	0.9433	0.9433		0.04	0.5425	0.6202		-0.05	0.8794	0.908	
	t(15;17) vs the rest	0.09	0.1864	0.3286		0.07	0.2609	0.3083		0.13	0.4258	0.554	
	inv(16) vs the rest	-0.11	0.0027	0.005	**	0.01	0.8757	0.94		-0.38	0.029	0.047	*
	Intermediate Risk Cytogenetics vs the rest	0.07	0.0169	0.1465		0.01	0.8006	0.8006		0.1	0.6504	0.89	
	Complex Cytogenetics vs the rest	-0.04	0.7412	0.9993		0.04	0.761	0.8156		0.13	0.477	0.84	
	Poor Risk Cytogenetics vs the rest	-0.03	0.5095	0.7359		-0.03	0.6436	0.9675		0.29	0.1335	0.647	
DUOX2	Normal karyotype vs the rest					-0.07	0.1176	0.392		-0.19	0.3748	0.577	
	t(8;21) vs the rest					0.21	0.1031	0.3348		1.06	0.2706	0.352	
	t(15;17) vs the rest					0.08	0.1955	0.2824		-0.09	0.7864	0.852	
	inv(16) vs the rest	NA				-0.02	0.7507	0.94		-0.95	0.0072	0.016	*
	Intermediate Risk Cytogenetics vs the rest					-0.05	0.2514	0.3268		0.14	0.6845	0.89	
	Complex Cytogenetics vs the rest					0.07	0.4266	0.7886		0.12	0.7114	0.925	
	Poor Risk Cytogenetics vs the rest					0	0.989	0.989		0.37	0.3017	0.647	
CYBA	Normal karyotype vs the rest	0.18	0.0348	0.2262		0.06	0.5514	0.6517		0.14	0.2191	0.434	
	t(8;21) vs the rest	-0.16	0.1312	0.2843		0.2	0.1428	0.3348		-0.19	0.3922	0.464	
	t(15;17) vs the rest	-0.22	0.237	0.3423		-0.31	0.0348	0.0996		0.32	0.0563	0.146	
	inv(16) vs the rest	0.47	<0.001	<0.001	***	0.3	0.0215	0.0699		0.21	0.1523	0.198	
	Intermediate Risk Cytogenetics vs the rest	-0.12	0.2934	0.4134		0.2	0.1068	0.1543		-0.02	0.9153	0.944	
	Complex Cytogenetics vs the rest	-0.46	0.214	0.606		-0.76	0.0019	0.0247	*	-0.34	0.0453	0.589	
	Poor Risk Cytogenetics vs the rest	-0.27	0.0835	0.4416		0.26	0.1491	0.7744		-0.26	0.2817	0.647	
NCF1	Normal karyotype vs the rest	0.55	0.0075	0.0975		0.37	0.1239	0.392		0.68	0.0709	0.371	
	t(8;21) vs the rest	**-1.27**	<0.001	<0.001	***	**-1.47**	<0.001	<0.001	***	**-2.64**	<0.001	<0.001	***
	t(15;17) vs the rest	**-2.06**	<0.001	<0.001	***	**-1.84**	<0.001	<0.001	***	**-2.39**	<0.001	<0.001	***
	inv(16) vs the rest	**2.44**	<0.001	<0.001	***	**1.76**	<0.001	0.0013	**	**2.92**	<0.001	<0.001	***
	Intermediate Risk Cytogenetics vs the rest	-0.56	0.0338	0.1465		0.51	0.1031	0.1543		0.33	0.5172	0.89	
	Complex Cytogenetics vs the rest	-0.1	0.8349	0.9993		-0.17	0.6066	0.7886		-0.4	0.3587	0.84	
	Poor Risk Cytogenetics vs the rest	-0.35	0.2005	0.4752		0.45	0.3764	0.9675		-0.12	0.8322	0.902	
NCF2	Normal karyotype vs the rest	0.38	0.2801	0.4603		0.23	0.4393	0.5795		0.18	0.5817	0.688	
	t(8;21) vs the rest	-0.56	0.2128	0.3458		-0.9	0.025	0.1083		**-1.59**	<0.001	<0.001	***
	t(15;17) vs the rest	-3.35	<0.001	<0.001	***	**-2.4**	<0.001	<0.001	***	**-1.79**	<0.001	0.001	**
	inv(16) vs the rest	3.11	<0.001	<0.001	***	**2.16**	<0.001	<0.001	***	**1.68**	<0.001	<0.001	***
	Intermediate Risk Cytogenetics vs the rest	-0.91	0.0606	0.1576		0.72	0.047	0.0873		0.62	0.1666	0.702	
	Complex Cytogenetics vs the rest	0	0.9993	0.9993		0.33	0.4727	0.7886		0.09	0.814	0.936	

	**Poor Risk Cytogenetics vs the rest**	0.12	0.8161	0.9204		0.2	0.7103	0.9675		-0.01	0.9793	0.979	
	**Normal karyotype vs the rest**	0.06	0.5632	0.6174		0.15	0.2412	0.392		0.1	0.3994	0.577	
	**t(8;21) vs the rest**	0.2	0.0284	0.0923		0.31	0.2109	0.3427		0.03	0.9078	0.908	
	**t(15;17) vs the rest**	-0.25	0.2022	0.3286		-0.65	0.0016	0.0069	**	0.15	0.2897	0.47	
NCF4	**inv(16) vs the rest**	0.37	<0.001	<0.001	***	-0.05	0.7784	0.94		-0.13	0.3548	0.384	
	**Intermediate Risk Cytogenetics vs the rest**	-0.24	0.057	0.1576		0.07	0.6633	0.7186		-0.28	0.1645	0.702	
	**Complex Cytogenetics vs the rest**	-0.07	0.8694	0.9993		0.14	0.5401	0.7886		0.11	0.701	0.925	
	**Poor Risk Cytogenetics vs the rest**	-0.09	0.6206	0.8068		0.14	0.6141	0.9675		-0.16	0.3981	0.647	
	**Normal karyotype vs the rest**	0.03	0.167	0.4435		0.15	0.0276	0.3588		0.43	0.1142	0.371	
	**t(8;21) vs the rest**	-0.06	0.0526	0.1368		-0.17	0.1995	0.3427		-2.7	0.0026	0.007	**
	**t(15;17) vs the rest**	-0.02	0.6869	0.6869		-0.01	0.9426	0.9426		0.07	0.863	0.863	
NOXA1	**inv(16) vs the rest**	-0.09	<0.001	0.0011	**	-0.15	0.1509	0.327		-1.74	0.0032	0.008	**
	**Intermediate Risk Cytogenetics vs the rest**	0.04	0.3174	0.4134		-0.16	0.0434	0.0873		-0.28	0.4764	0.89	
	**Complex Cytogenetics vs the rest**	-0.08	<0.001	<0.001	***	0.14	0.5624	0.7886		0.67	0.1302	0.84	
	**Poor Risk Cytogenetics vs the rest**	0	0.8921	0.9204		-0.06	0.6684	0.9675		0.45	0.1814	0.647	
	**Normal karyotype vs the rest**					0.03	0.4458	0.5795		0.01	0.9568	0.957	
	**t(8;21) vs the rest**					0.01	0.897	0.897		-0.48	0.0972	0.181	
	**t(15;17) vs the rest**					0.01	0.9373	0.9426		0.22	0.3253	0.47	
NOXO1	**inv(16) vs the rest**		NA			-0.04	0.5111	0.94		-0.53	0.0995	0.144	
	**Intermediate Risk Cytogenetics vs the rest**					-0.13	0.0039	0.0464	*	0.08	0.6618	0.89	
	**Complex Cytogenetics vs the rest**					0.18	0.2077	0.7886		0.03	0.8637	0.936	
	**Poor Risk Cytogenetics vs the rest**					-0.01	0.9314	0.989		0.16	0.457	0.659	

Student's t-test was used for pairwise comparisons. q-values: adjusted p-values calculated using the BH method.

For GSE6891 dataset, NOX1, NOX3, NOX4, NOX5, DUOX2, and NOXO1 were omitted because they are mostly not expressed and showed 0 variance for at least one comparison.

Table S5. Univariate cox regression analysis of OS and EFS in 3 AML datasets.

A) OS table

	**GSE6891 (OS N = 279)**			**GSE10385 (OS N = 223)**			**TCGA (OS N = 173)**		
**Variable**	**Hazard Ratio**	**95% CI**	**Wald's p-value**	**Hazard Ratio**	**95% CI**	**Wald's p-value**	**Hazard Ratio**	**95% CI**	**Wald's p-value**
NOX1 (High vs Low)	0.67	(0.33-1.37)	0.277	0.84	(0.58-1.21)	0.358	0.96	(0.66-1.38)	0.814
CYBB (High vs Low)	1.05	(0.77-1.42)	0.759	**1.48**	(1.02-2.13)	**0.037**	1.3	(0.9-1.88)	0.166
NOX3 (High vs Low)*	-	-	-	**0.65**	(0.45-0.94)	**0.021**	-	-	-
NOX4 (High vs Low)*	-	-	-	1.04	(0.72-1.49)	0.848	-	-	-
NOX5 (High vs Low)	0.77	(0.45-1.32)	0.343	0.7	(0.48-1.01)	0.053	0.94	(0.65-1.36)	0.744
DUOX1 (High vs Low)	0.98	(0.69-1.4)	0.929	0.86	(0.6-1.24)	0.427	0.67	(0.46-0.96)	0.031
DUOX2 (High vs Low)	-	-	-	1.11	(0.77-1.6)	0.566	0.97	(0.67-1.4)	0.882
CYBA (High vs Low)	0.89	(0.66-1.21)	0.452	1.11	(0.77-1.6)	0.575	1.04	(0.72-1.5)	0.843
*NCF1 (*High vs Low)	0.98	(0.72-1.32)	0.874	**1.76**	(1.22-2.54)	**0.003**	1.4	(0.97-2.02)	0.075
NCF2 (High vs Low)	0.92	(0.68-1.25)	0.596	**1.51**	(1.05-2.18)	**0.026**	1.25	(0.87-1.81)	0.231
NCF4 (High vs Low)	1.04	(0.77-1.41)	0.806	**2.27**	(1.56-3.3)	**<0.001**	**1.37**	(0.95-1.98)	**0.094**
NOXA1 (High vs Low)	-	-	-	1.04	(0.72-1.49)	0.837	1.13	(0.78-1.63)	0.514
NOXO1 (High vs Low)	-	-	-	0.88	(0.61-1.26)	0.474	0.98	(0.68-1.41)	0.899

B) EFS table

	**GSE6891 (EFS N = 279)**			**GSE10385 (EFS N = 223)**			**TCGA (EFS N = 173)**		
**Variable**	**Hazard Ratio**	**95% CI**	**Wald's p-value**	**Hazard Ratio**	**95% CI**	**Wald's p-value**	**Hazard Ratio**	**95% CI**	**Wald's p-value**
NOX1 (High vs Low)	0.63	(0.32-1.23)	0.176	0.9	(0.65-1.25)	0.534	1.02	(0.73-1.44)	0.896
CYBB (High vs Low)	1.07	(0.8-1.42)	0.669	**1.46**	(1.05-2.04)	**0.025**	1.27	(0.9-1.79)	0.171
NOX3 (High vs Low)	-	-	-	**0.69**	(0.5-0.96)	**0.03**	-	-	-
NOX4 (High vs Low)	-	-	-	1	(0.72-1.39)	0.985	-	-	-
NOX5 (High vs Low)	0.78	(0.47-1.28)	0.319	0.84	(0.61-1.17)	0.307	1.04	(0.74-1.46)	0.837
DUOX1 (High vs Low)	0.87	(0.62-1.22)	0.413	0.84	(0.61-1.17)	0.312	0.66	(0.47-0.93)	0.018
DUOX2 (High vs Low)	-	-	-	0.93	(0.67-1.3)	0.676	0.91	(0.65-1.28)	0.59
CYBA (High vs Low)	0.81	(0.6-1.08)	0.147	1.13	(0.81-1.56)	0.484	1	(0.71-1.4)	0.983
NCF1 (High vs Low)	0.86	(0.64-1.14)	0.296	**1.78**	(1.27-2.49)	**0.001**	1.31	(0.93-1.84)	0.126
NCF2 (High vs Low)	0.87	(0.65-1.16)	0.349	**1.43**	(1.03-2)	**0.033**	1.24	(0.88-1.75)	0.212
NCF4 (High vs Low)	0.99	(0.74-1.33)	0.96	**2.53**	(1.8-3.58)	**<0.001**	**1.56**	(1.11-2.2)	**0.011**
NOXA1 (High vs Low)	-	-	-	1.02	(0.74-1.42)	0.895	1.21	(0.86-1.7)	0.282
NOXO1 (High vs Low)	-	-	-	0.86	(0.62-1.2)	0.374	1.1	(0.78-1.55)	0.583

NOX3, NOX4, DUOX2 and NOXO1 were mostly not expressed in the GSE6891 dataset; NOX3 and NOX4 were mostly not expressed in the TCGA dataset. NOXA1 violated proportional hazard's assumption in the GSE6891 dataset.

Table S6. Cox regression analysis of NOX in 3 AML datasets after adjustment for age and cytogenetic abnormalities.

**A) GSE6891 dataset**		**GSE6891 (OS N = 243)**			**GSE6891 (EFS N = 243)**		
**Multivariate model**	**Variable**	**Hazard Ratio**	**95% CI**	**P***	**Hazard Ratio**	**95% CI**	**P***
NOX1	Cytogenetically Normal	1	-	-	1	-	-
	t(15;17)	NA	NA	NA	NA	NA	NA
	t(8;21)	0.59	(0.29-1.19)	0.14	0.53	(0.27-1.04)	0.064
	inv(16)	0.66	(0.33-1.34)	0.254	0.78	(0.42-1.46)	0.437
	Intermediate Risk Cytogenetics	1.01	(0.66-1.56)	0.948	0.9	(0.59-1.37)	0.631
	Complex Cytogenetics	1.82	(0.79-4.22)	0.16	1.7	(0.74-3.93)	0.212
	Poor Risk Cytogenetics	1.34	(0.78-2.28)	0.292	1.32	(0.79-2.19)	0.286
	Age	1.01	(1-1.02)	0.195	1	(0.99-1.02)	0.591
	***NOX1* (High vs Low)**	0.64	(0.31-1.33)	0.232	0.53	(0.26-1.09)	0.082
CYBB	Cytogenetically Normal	1	-	-	1	-	-
	t(15;17)	NA	NA	NA	NA	NA	NA
	t(8;21)	0.61	(0.3-1.25)	0.178	0.56	(0.28-1.1)	0.091
	inv(16)	0.65	(0.31-1.34)	0.244	0.77	(0.4-1.46)	0.42
	Intermediate Risk Cytogenetics	1.06	(0.7-1.63)	0.774	0.96	(0.64-1.46)	0.862
	Complex Cytogenetics	1.87	(0.81-4.32)	0.144	1.75	(0.76-4.04)	0.19
	Poor Risk Cytogenetics	1.36	(0.79-2.32)	0.268	1.34	(0.81-2.23)	0.253
	Age	1.01	(0.99-1.02)	0.268	1	(0.99-1.02)	0.766
	***CYBB* (High vs Low)**	1.03	(0.73-1.45)	0.867	1	(0.72-1.4)	0.979
NOX3	Cytogenetically Normal	NA			NA		
	t(15;17)						
	t(8;21)						
	inv(16)						
	Intermediate Risk Cytogenetics						
	Complex Cytogenetics						
	Poor Risk Cytogenetics						
	Age						
	***NOX3* (High vs Low)**						
NOX4	Cytogenetically Normal	NA			NA		
	t(15;17)						
	t(8;21)						
	inv(16)						

	Intermediate Risk Cytogenetics						
	Complex Cytogenetics						
	Poor Risk Cytogenetics						
	Age						
	***NOX4* (High vs Low)**						
NOX5	Cytogenetically Normal	1	-	-	1	-	-
	t(15;17)	NA	NA	NA	NA	NA	NA
	t(8;21)	0.61	(0.3-1.25)	0.176	0.56	(0.29-1.09)	0.089
	inv(16)	0.66	(0.33-1.35)	0.258	0.77	(0.41-1.44)	0.412
	Intermediate Risk Cytogenetics	1.06	(0.69-1.62)	0.797	0.96	(0.64-1.46)	0.858
	Complex Cytogenetics	1.86	(0.8-4.3)	0.149	1.75	(0.76-4.04)	0.192
	Poor Risk Cytogenetics	1.35	(0.79-2.31)	0.275	1.34	(0.81-2.23)	0.255
	Age	1.01	(0.99-1.02)	0.242	1	(0.99-1.02)	0.757
	***NOX5* (High vs Low)**	0.92	(0.52-1.65)	0.789	0.99	(0.57-1.69)	0.961
DUOX1	Cytogenetically Normal	1	-	-	1	-	-
	t(15;17)	NA	NA	NA	NA	NA	NA
	t(8;21)	0.6	(0.3-1.22)	0.159	0.56	(0.29-1.1)	0.093
	inv(16)	0.66	(0.32-1.33)	0.245	0.77	(0.41-1.43)	0.404
	Intermediate Risk Cytogenetics	1.05	(0.69-1.61)	0.813	0.97	(0.64-1.47)	0.887
	Complex Cytogenetics	1.9	(0.82-4.42)	0.134	1.72	(0.74-3.98)	0.204
	Poor Risk Cytogenetics	1.37	(0.8-2.35)	0.255	1.33	(0.8-2.22)	0.265
	Age	1.01	(0.99-1.02)	0.249	1	(0.99-1.02)	0.763
	***DUOX1* (High vs Low)**	1.09	(0.75-1.6)	0.648	0.92	(0.63-1.33)	0.656
DUOX2	Cytogenetically Normal						
	t(15;17)						
	t(8;21)						
	inv(16)						
	Intermediate Risk Cytogenetics		NA			NA	
	Complex Cytogenetics						
	Poor Risk Cytogenetics						
	Age						
	***DUOX2* (High vs Low)**						
CYBA	Cytogenetically Normal	1	-	-	1	-	-
	t(15;17)	NA	NA	NA	NA	NA	NA
	t(8;21)	0.6	(0.29-1.21)	0.153	0.53	(0.27-1.04)	0.067
	inv(16)	0.66	(0.33-1.34)	0.253	0.78	(0.42-1.46)	0.445
	Intermediate Risk Cytogenetics	1.04	(0.68-1.6)	0.848	0.93	(0.61-1.41)	0.726

	Complex Cytogenetics	1.82	(0.78-4.23)	0.163	1.65	(0.71-3.82)	0.243
	Poor Risk Cytogenetics	1.32	(0.77-2.28)	0.309	1.28	(0.77-2.14)	0.337
	Age	1.01	(0.99-1.02)	0.247	1	(0.99-1.02)	0.716
	***CYBA* (High vs Low)**	0.89	(0.64-1.24)	0.493	0.81	(0.59-1.12)	0.196
NCF1	Cytogenetically Normal	1	-	-	1	-	-
	t(15;17)	NA	NA	NA	NA	NA	NA
	t(8;21)	0.61	(0.3-1.24)	0.17	0.54	(0.28-1.06)	0.073
	inv(16)	0.66	(0.32-1.36)	0.257	0.82	(0.43-1.55)	0.533
	Intermediate Risk Cytogenetics	1.06	(0.69-1.63)	0.779	0.94	(0.62-1.43)	0.783
	Complex Cytogenetics	1.87	(0.81-4.33)	0.143	1.71	(0.74-3.96)	0.209
	Poor Risk Cytogenetics	1.35	(0.79-2.32)	0.268	1.35	(0.81-2.23)	0.251
	Age	1.01	(0.99-1.02)	0.256	1	(0.99-1.02)	0.701
	***NCF1* (High vs Low)**	1	(0.71-1.41)	0.981	0.88	(0.63-1.22)	0.426
NCF2	Cytogenetically Normal	1	-	-	1	-	-
	t(15;17)	NA	NA	NA	NA	NA	NA
	t(8;21)	0.6	(0.3-1.21)	0.154	0.54	(0.28-1.06)	0.073
	inv(16)	0.69	(0.34-1.42)	0.312	0.81	(0.43-1.53)	0.518
	Intermediate Risk Cytogenetics	1.05	(0.68-1.6)	0.836	0.94	(0.62-1.43)	0.779
	Complex Cytogenetics	1.87	(0.81-4.33)	0.143	1.7	(0.74-3.94)	0.213
	Poor Risk Cytogenetics	1.35	(0.79-2.32)	0.27	1.33	(0.8-2.2)	0.277
	Age	1.01	(0.99-1.02)	0.217	1	(0.99-1.02)	0.651
	***NCF2* (High vs Low)**	0.88	(0.63-1.24)	0.47	0.85	(0.61-1.18)	0.327
NCF4	Cytogenetically Normal	1	-	-	1	-	-
	t(15;17)	NA	NA	NA	NA	NA	NA
	t(8;21)	0.61	(0.3-1.23)	0.167	0.56	(0.29-1.09)	0.087
	inv(16)	0.66	(0.32-1.34)	0.246	0.77	(0.41-1.43)	0.404
	Intermediate Risk Cytogenetics	1.06	(0.69-1.62)	0.78	0.96	(0.64-1.46)	0.859
	Complex Cytogenetics	1.87	(0.81-4.32)	0.144	1.74	(0.76-4.03)	0.192
	Poor Risk Cytogenetics	1.36	(0.79-2.33)	0.266	1.35	(0.81-2.24)	0.251
	Age	1.01	(0.99-1.02)	0.252	1	(0.99-1.02)	0.758
	***NCF4* (High vs Low)**	0.98	(0.71-1.37)	0.925	0.98	(0.72-1.34)	0.902
NOXA1	Cytogenetically Normal		NA			NA	
	t(15;17)						
	t(8;21)						
	inv(16)						
	Intermediate Risk Cytogenetics						
	Complex Cytogenetics						

	Poor Risk Cytogenetics						
	Age						
	***NOXA1* (High vs Low)**						
NOXO1	Cytogenetically Normal	NA			NA		
	t(15;17)						
	t(8;21)						
	inv(16)						
	Intermediate Risk Cytogenetics						
	Complex Cytogenetics						
	Poor Risk Cytogenetics						
	Age						
	***NOXO1* (High vs Low)**						

**B) GSE10358 dataset**		**GSE10358 (OS N = 220)**			**GSE10358 (EFS N = 220)**		
**Multivariate model**	**Variable**	**Hazard Ratio**	**95% CI**	**P***	**Hazard Ratio**	**95% CI**	**P***
NOX1	Cytogenetically Normal	1	-	-	1	-	-
	t(15;17)	0.17	(0.06-0.48)	0.001	0.24	(0.11-0.52)	<0.001
	t(8;21)	0.88	(0.4-1.93)	0.743	0.7	(0.32-1.53)	0.373
	inv(16)	0.24	(0.08-0.78)	0.018	0.34	(0.14-0.86)	0.022
	Intermediate Risk Cytogenetics	1.35	(0.84-2.18)	0.209	1.32	(0.86-2.04)	0.203
	Complex Cytogenetics	2.37	(1.19-4.7)	0.014	1.6	(0.82-3.11)	0.167
	Poor Risk Cytogenetics	1.1	(0.54-2.25)	0.79	1.2	(0.63-2.29)	0.579
	Age	1.03	(1.01-1.04)	<0.001	1.02	(1.01-1.03)	0.001
	***NOX1* (High vs Low)**	1.18	(0.79-1.78)	0.414	1.18	(0.82-1.71)	0.367
CYBB	Cytogenetically Normal	1	-	-	1	-	-
	t(15;17)	0.17	(0.06-0.47)	0.001	0.23	(0.1-0.51)	<0.001
	t(8;21)	1.1	(0.48-2.49)	0.827	0.86	(0.38-1.94)	0.72
	inv(16)	0.2	(0.06-0.66)	0.008	0.3	(0.12-0.75)	0.01
	Intermediate Risk Cytogenetics	1.25	(0.79-2)	0.341	1.25	(0.82-1.92)	0.305
	Complex Cytogenetics	2.41	(1.24-4.69)	0.01	1.63	(0.85-3.13)	0.142
	Poor Risk Cytogenetics	1.14	(0.56-2.33)	0.715	1.25	(0.65-2.38)	0.502
	Age	1.02	(1.01-1.03)	0.002	1.02	(1-1.03)	0.008
	***CYBB* (High vs Low)**	1.49	(0.99-2.26)	0.058	1.41	(0.97-2.05)	0.074
NOX3	Cytogenetically Normal	1	-	-	1	-	-
	t(15;17)	0.17	(0.06-0.47)	0.001	0.23	(0.1-0.51)	<0.001
	t(8;21)	0.89	(0.4-1.96)	0.776	0.71	(0.33-1.56)	0.399
	inv(16)	0.22	(0.07-0.72)	0.012	0.32	(0.13-0.79)	0.014

	Intermediate Risk Cytogenetics	1.26	(0.79-2.01)	0.331	1.23	(0.8-1.89)	0.342
	Complex Cytogenetics	2.03	(1.04-3.95)	0.037	1.4	(0.73-2.67)	0.311
	Poor Risk Cytogenetics	1.12	(0.55-2.29)	0.749	1.2	(0.63-2.3)	0.576
	Age	1.02	(1.01-1.04)	<0.001	1.02	(1.01-1.03)	0.001
	***NOX3* (High vs Low)**	0.71	(0.49-1.03)	0.073	0.73	(0.52-1.02)	0.068
NOX4	Cytogenetically Normal	1	-	-	1	-	-
	t(15;17)	0.17	(0.06-0.46)	0.001	0.23	(0.1-0.5)	<0.001
	t(8;21)	0.88	(0.4-1.94)	0.755	0.71	(0.33-1.55)	0.391
	inv(16)	0.23	(0.07-0.74)	0.013	0.33	(0.13-0.82)	0.017
	Intermediate Risk Cytogenetics	1.3	(0.81-2.07)	0.276	1.27	(0.83-1.95)	0.277
	Complex Cytogenetics	2.1	(1.08-4.09)	0.028	1.44	(0.75-2.75)	0.271
	Poor Risk Cytogenetics	1.02	(0.5-2.1)	0.956	1.14	(0.59-2.19)	0.7
	Age	1.03	(1.01-1.04)	<0.001	1.02	(1.01-1.03)	0.001
	***NOX4* (High vs Low)**	1.24	(0.86-1.81)	0.252	1.16	(0.82-1.63)	0.405
NOX5	Cytogenetically Normal	1	-	-	1	-	-
	t(15;17)	0.17	(0.06-0.47)	0.001	0.23	(0.11-0.51)	<0.001
	t(8;21)	0.89	(0.4-1.96)	0.772	0.72	(0.33-1.57)	0.405
	inv(16)	0.23	(0.07-0.73)	0.013	0.34	(0.13-0.85)	0.021
	Intermediate Risk Cytogenetics	1.24	(0.77-2)	0.376	1.29	(0.83-2)	0.26
	Complex Cytogenetics	2.03	(1.03-4.01)	0.041	1.49	(0.77-2.91)	0.24
	Poor Risk Cytogenetics	1.05	(0.51-2.15)	0.902	1.19	(0.62-2.29)	0.595
	Age	1.02	(1.01-1.04)	<0.001	1.02	(1.01-1.03)	0.001
	***NOX5* (High vs Low)**	0.83	(0.56-1.23)	0.356	1.01	(0.71-1.45)	0.945
DUOX1	Cytogenetically Normal	1	-	-	1	-	-
	t(15;17)	0.17	(0.06-0.47)	0.001	0.23	(0.11-0.51)	<0.001
	t(8;21)	0.89	(0.41-1.97)	0.779	0.72	(0.33-1.56)	0.402
	inv(16)	0.24	(0.08-0.78)	0.018	0.34	(0.14-0.85)	0.02
	Intermediate Risk Cytogenetics	1.31	(0.82-2.09)	0.251	1.29	(0.84-1.97)	0.247
	Complex Cytogenetics	2.26	(1.16-4.4)	0.016	1.5	(0.79-2.87)	0.217
	Poor Risk Cytogenetics	1.09	(0.53-2.22)	0.817	1.19	(0.62-2.27)	0.601
	Age	1.03	(1.01-1.04)	<0.001	1.02	(1.01-1.03)	0.001
	***DUOX1* (High vs Low)**	1.13	(0.77-1.66)	0.526	1.07	(0.76-1.51)	0.707
DUOX2	Cytogenetically Normal	1	-	-	1	-	-
	t(15;17)	0.17	(0.06-0.47)	0.001	0.23	(0.11-0.52)	<0.001
	t(8;21)	0.85	(0.38-1.9)	0.694	0.74	(0.33-1.62)	0.447
	inv(16)	0.24	(0.08-0.79)	0.018	0.34	(0.13-0.84)	0.019
	Intermediate Risk Cytogenetics	1.3	(0.82-2.07)	0.266	1.29	(0.84-1.98)	0.246

	Complex Cytogenetics	2.17	(1.12-4.21)	0.021	1.49	(0.78-2.83)	0.226
	Poor Risk Cytogenetics	1.07	(0.52-2.19)	0.849	1.21	(0.63-2.32)	0.567
	Age	1.02	(1.01-1.04)	<0.001	1.02	(1.01-1.03)	0.001
	***DUOX2* (High vs Low)**	1.15	(0.79-1.67)	0.467	0.93	(0.67-1.31)	0.698
CYBA	Cytogenetically Normal	1	-	-	1	-	-
	t(15;17)	0.17	(0.06-0.49)	0.001	0.23	(0.11-0.52)	<0.001
	t(8;21)	0.9	(0.41-1.99)	0.801	0.72	(0.33-1.58)	0.414
	inv(16)	0.24	(0.07-0.77)	0.016	0.34	(0.13-0.84)	0.02
	Intermediate Risk Cytogenetics	1.31	(0.82-2.09)	0.256	1.28	(0.84-1.97)	0.252
	Complex Cytogenetics	2.26	(1.14-4.48)	0.019	1.5	(0.78-2.91)	0.228
	Poor Risk Cytogenetics	1.09	(0.53-2.23)	0.811	1.19	(0.62-2.27)	0.604
	Age	1.02	(1.01-1.04)	<0.001	1.02	(1.01-1.03)	0.001
	***CYBA* (High vs Low)**	1.06	(0.72-1.56)	0.764	1.03	(0.73-1.46)	0.862
NCF1	Cytogenetically Normal	1	-	-	1	-	-
	t(15;17)	0.19	(0.07-0.54)	0.002	0.26	(0.12-0.59)	0.001
	t(8;21)	0.98	(0.44-2.19)	0.962	0.8	(0.36-1.77)	0.583
	inv(16)	0.22	(0.07-0.71)	0.012	0.3	(0.12-0.76)	0.011
	Intermediate Risk Cytogenetics	1.27	(0.8-2.03)	0.308	1.25	(0.81-1.91)	0.309
	Complex Cytogenetics	2.22	(1.15-4.28)	0.018	1.49	(0.78-2.83)	0.226
	Poor Risk Cytogenetics	1.07	(0.53-2.19)	0.843	1.18	(0.62-2.25)	0.616
	Age	1.02	(1.01-1.04)	0.001	1.02	(1.01-1.03)	0.003
	***NCF1* (High vs Low)**	1.27	(0.85-1.88)	0.242	1.33	(0.92-1.91)	0.131
NCF2	Cytogenetically Normal	1	-	-	1	-	-
	t(15;17)	0.18	(0.07-0.51)	0.001	0.24	(0.11-0.54)	<0.001
	t(8;21)	0.98	(0.44-2.18)	0.951	0.76	(0.34-1.68)	0.494
	inv(16)	0.22	(0.07-0.7)	0.011	0.32	(0.12-0.8)	0.015
	Intermediate Risk Cytogenetics	1.29	(0.81-2.06)	0.279	1.28	(0.83-1.96)	0.261
	Complex Cytogenetics	2.2	(1.14-4.26)	0.019	1.48	(0.78-2.82)	0.23
	Poor Risk Cytogenetics	1.04	(0.51-2.14)	0.906	1.16	(0.61-2.23)	0.645
	Age	1.02	(1.01-1.04)	<0.001	1.02	(1.01-1.03)	0.002
	***NCF2* (High vs Low)**	1.25	(0.85-1.85)	0.262	1.14	(0.8-1.63)	0.468
NCF4	Cytogenetically Normal	1	-	-	1	-	-
	t(15;17)	0.2	(0.07-0.57)	0.002	0.29	(0.13-0.63)	0.002
	t(8;21)	0.89	(0.41-1.96)	0.777	0.71	(0.32-1.55)	0.387
	inv(16)	0.26	(0.08-0.84)	0.024	0.4	(0.16-0.99)	0.049
	Intermediate Risk Cytogenetics	1.25	(0.79-1.99)	0.348	1.24	(0.81-1.89)	0.322
	Complex Cytogenetics	1.85	(0.95-3.61)	0.07	1.23	(0.64-2.35)	0.533

	Poor Risk Cytogenetics	1.23	(0.6-2.52)	0.567	1.34	(0.7-2.56)	0.375
	Age	1.03	(1.01-1.04)	<0.001	1.02	(1.01-1.03)	<0.001
	***NCF4* (High vs Low)**	**1.96**	(1.32-2.89)	**0.001**	**2.18**	(1.53-3.12)	**<0.001**
NOXA1	Cytogenetically Normal	1	-	-	1	-	-
	t(15;17)	0.17	(0.06-0.48)	0.001	0.23	(0.11-0.52)	<0.001
	t(8;21)	0.89	(0.4-2)	0.784	0.73	(0.33-1.61)	0.439
	inv(16)	0.24	(0.07-0.78)	0.018	0.35	(0.14-0.87)	0.024
	Intermediate Risk Cytogenetics	1.31	(0.81-2.09)	0.268	1.3	(0.84-2.01)	0.238
	Complex Cytogenetics	2.2	(1.13-4.28)	0.02	1.47	(0.77-2.8)	0.246
	Poor Risk Cytogenetics	1.09	(0.53-2.23)	0.81	1.21	(0.63-2.32)	0.573
	Age	1.02	(1.01-1.04)	<0.001	1.02	(1.01-1.03)	0.001
	***NOXA1* (High vs Low)**	1	(0.67-1.48)	0.992	1.06	(0.74-1.51)	0.761
NOXO1	Cytogenetically Normal	1	-	-	1	-	-
	t(15;17)	0.17	(0.06-0.47)	0.001	0.23	(0.11-0.51)	<0.001
	t(8;21)	0.89	(0.41-1.97)	0.783	0.72	(0.33-1.57)	0.404
	inv(16)	0.24	(0.07-0.77)	0.017	0.34	(0.14-0.85)	0.021
	Intermediate Risk Cytogenetics	1.3	(0.81-2.11)	0.279	1.29	(0.83-2.01)	0.258
	Complex Cytogenetics	2.2	(1.14-4.26)	0.019	1.48	(0.78-2.82)	0.23
	Poor Risk Cytogenetics	1.09	(0.53-2.23)	0.813	1.2	(0.62-2.31)	0.592
	Age	1.02	(1.01-1.04)	<0.001	1.02	(1.01-1.03)	0.001
	***NOXO1* (High vs Low)**	0.99	(0.68-1.45)	0.973	1.02	(0.72-1.45)	0.917

**C) TCGA dataset**		**TCGA (OS N = 170)**			**TCGA (EFS N = 170)**		
**Multivariate model**	**Variable**	**Hazard Ratio**	**95% CI**	**P***	**Hazard Ratio**	**95% CI**	**P***
NOX1	Cytogenetically Normal	1	-	-	1	-	-
	t(15;17)	0.51	(0.17-1.57)	0.242	0.64	(0.23-1.79)	0.398
	t(8;21)	0.54	(0.1-2.89)	0.474	0.55	(0.11-2.8)	0.471
	inv(16)	0.32	(0.08-1.23)	0.097	0.4	(0.12-1.28)	0.123
	Intermediate Risk Cytogenetics	1.04	(0.48-2.26)	0.918	1.15	(0.55-2.44)	0.707
	Complex Cytogenetics	3.56	(1.45-8.75)	0.006	3	(1.3-6.91)	0.01
	Poor Risk Cytogenetics	1.63	(0.68-3.9)	0.274	2.85	(1.14-7.09)	0.025
	Age	stratifier	stratifier	stratifier	stratifier	stratifier	stratifier
	***NOX1* (High vs Low)**	1.6	(0.92-2.77)	0.095	1.21	(0.73-1.99)	0.467
CYBB	Cytogenetically Normal	1	-	-	1	-	-
	t(15;17)	0.49	(0.16-1.52)	0.215	0.64	(0.23-1.78)	0.391
	t(8;21)	0.65	(0.12-3.47)	0.61	0.61	(0.12-3.16)	0.556
	inv(16)	0.4	(0.11-1.53)	0.181	0.41	(0.12-1.33)	0.136

	Intermediate Risk Cytogenetics	1.16	(0.53-2.52)	0.715	1.15	(0.53-2.49)	0.723
	Complex Cytogenetics	3.92	(1.61-9.57)	0.003	3.18	(1.4-7.23)	0.006
	Poor Risk Cytogenetics	1.7	(0.72-4.03)	0.227	2.95	(1.19-7.31)	0.02
	Age	stratifier	stratifier	stratifier	stratifier	stratifier	stratifier
	***CYBB* (High vs Low)**	0.96	(0.54-1.7)	0.882	1.06	(0.62-1.83)	0.822
NOX3	Cytogenetically Normal						
	t(15;17)						
	t(8;21)						
	inv(16)						
	Intermediate Risk Cytogenetics		NA			NA	
	Complex Cytogenetics						
	Poor Risk Cytogenetics						
	Age						
	***NOX3* (High vs Low)**						
NOX4	Cytogenetically Normal						
	t(15;17)						
	t(8;21)						
	inv(16)						
	Intermediate Risk Cytogenetics		NA			NA	
	Complex Cytogenetics						
	Poor Risk Cytogenetics						
	Age						
	***NOX4* (High vs Low)**						
NOX5	Cytogenetically Normal	1	-	-	1	-	-
	t(15;17)	0.49	(0.16-1.53)	0.222	0.65	(0.23-1.81)	0.411
	t(8;21)	0.65	(0.12-3.56)	0.62	0.55	(0.11-2.91)	0.484
	inv(16)	0.39	(0.1-1.44)	0.158	0.4	(0.13-1.31)	0.13
	Intermediate Risk Cytogenetics	1.14	(0.52-2.46)	0.746	1.15	(0.54-2.45)	0.708
	Complex Cytogenetics	3.94	(1.61-9.66)	0.003	3.22	(1.41-7.38)	0.006
	Poor Risk Cytogenetics	1.71	(0.72-4.04)	0.221	2.93	(1.18-7.27)	0.021
	Age	stratifier	stratifier	stratifier	stratifier	stratifier	stratifier
	***NOX5* (High vs Low)**	0.98	(0.55-1.74)	0.935	0.91	(0.53-1.55)	0.727
DUOX1	Cytogenetically Normal	1	-	-	1	-	-
	t(15;17)	0.57	(0.18-1.79)	0.339	0.73	(0.25-2.07)	0.549
	t(8;21)	0.92	(0.16-5.16)	0.92	0.8	(0.15-4.25)	0.797
	inv(16)	0.4	(0.11-1.45)	0.163	0.39	(0.12-1.25)	0.112
	Intermediate Risk Cytogenetics	1.01	(0.46-2.24)	0.976	1.11	(0.51-2.39)	0.797

	Complex Cytogenetics	3.5	(1.43-8.57)	0.006	2.82	(1.23-6.44)	0.014
	Poor Risk Cytogenetics	2.03	(0.84-4.93)	0.116	3.3	(1.29-8.39)	0.012
	Age	stratifier	stratifier	stratifier	stratifier	stratifier	stratifier
	***DUOX1* (High vs Low)**	**0.48**	(0.26-0.86)	**0.014**	**0.46**	(0.26-0.8)	**0.007**
DUOX2	Cytogenetically Normal	1	-	-	1	-	-
	t(15;17)	0.49	(0.16-1.52)	0.219	0.65	(0.23-1.81)	0.409
	t(8;21)	0.67	(0.13-3.48)	0.636	0.62	(0.12-3.09)	0.557
	inv(16)	0.38	(0.1-1.4)	0.146	0.4	(0.13-1.27)	0.121
	Intermediate Risk Cytogenetics	1.13	(0.53-2.44)	0.751	1.18	(0.56-2.5)	0.669
	Complex Cytogenetics	3.89	(1.59-9.51)	0.003	3.11	(1.36-7.11)	0.007
	Poor Risk Cytogenetics	1.71	(0.72-4.03)	0.224	2.92	(1.18-7.24)	0.021
	Age	stratifier	stratifier	stratifier	stratifier	stratifier	stratifier
	***DUOX2* (High vs Low)**	0.88	(0.5-1.53)	0.649	0.8	(0.48-1.33)	0.39
CYBA	Cytogenetically Normal	1	-	-	1	-	-
	t(15;17)	0.45	(0.14-1.43)	0.179	0.58	(0.21-1.65)	0.307
	t(8;21)	0.67	(0.13-3.53)	0.634	0.63	(0.12-3.22)	0.583
	inv(16)	0.32	(0.09-1.21)	0.094	0.37	(0.12-1.18)	0.093
	Intermediate Risk Cytogenetics	1.14	(0.53-2.46)	0.73	1.17	(0.55-2.48)	0.68
	Complex Cytogenetics	4.14	(1.69-10.15)	0.002	3.37	(1.47-7.69)	0.004
	Poor Risk Cytogenetics	1.76	(0.75-4.17)	0.197	3.03	(1.22-7.5)	0.017
	Age	stratifier	stratifier	stratifier	stratifier	stratifier	stratifier
	***CYBA* (High vs Low)**	1.51	(0.84-2.72)	0.173	1.58	(0.93-2.69)	0.091
NCF1	Cytogenetically Normal	1	-	-	1	-	-
	t(15;17)	0.49	(0.15-1.54)	0.22	0.63	(0.22-1.75)	0.371
	t(8;21)	0.65	(0.12-3.46)	0.618	0.57	(0.11-2.91)	0.498
	inv(16)	0.4	(0.11-1.5)	0.175	0.44	(0.13-1.43)	0.171
	Intermediate Risk Cytogenetics	1.15	(0.53-2.48)	0.726	1.2	(0.56-2.57)	0.634
	Complex Cytogenetics	3.91	(1.6-9.57)	0.003	3.13	(1.37-7.16)	0.007
	Poor Risk Cytogenetics	1.72	(0.72-4.1)	0.22	2.99	(1.19-7.48)	0.02
	Age	stratifier	stratifier	stratifier	stratifier	stratifier	stratifier
	***NCF1* (High vs Low)**	0.97	(0.53-1.79)	0.922	0.91	(0.51-1.61)	0.736
NCF2	Cytogenetically Normal	1	-	-	1	-	-
	t(15;17)	0.46	(0.14-1.5)	0.199	0.65	(0.23-1.87)	0.424
	t(8;21)	0.61	(0.11-3.37)	0.569	0.6	(0.11-3.18)	0.551
	inv(16)	0.42	(0.11-1.58)	0.197	0.41	(0.13-1.34)	0.142
	Intermediate Risk Cytogenetics	1.16	(0.54-2.51)	0.699	1.17	(0.55-2.49)	0.687
	Complex Cytogenetics	3.91	(1.6-9.56)	0.003	3.17	(1.39-7.23)	0.006

	Poor Risk Cytogenetics	1.7	(0.72-4.01)	0.23	2.93	(1.18-7.27)	0.02
	Age	stratifier	stratifier	stratifier	stratifier	stratifier	stratifier
	***NCF2* (High vs Low)**	0.89	(0.48-1.65)	0.711	1.03	(0.58-1.83)	0.912
NCF4	Cytogenetically Normal	1	-	-	1	-	-
	t(15;17)	0.51	(0.16-1.59)	0.247	0.64	(0.23-1.8)	0.397
	t(8;21)	0.45	(0.08-2.51)	0.364	0.41	(0.08-2.16)	0.294
	inv(16)	0.4	(0.11-1.5)	0.176	0.48	(0.15-1.54)	0.218
	Intermediate Risk Cytogenetics	1.12	(0.52-2.44)	0.774	1.2	(0.56-2.57)	0.631
	Complex Cytogenetics	3.27	(1.31-8.16)	0.011	2.59	(1.11-6.01)	0.027
	Poor Risk Cytogenetics	1.64	(0.69-3.89)	0.263	2.84	(1.14-7.07)	0.025
	Age	stratifier	stratifier	stratifier	stratifier	stratifier	stratifier
	***NCF4* (High vs Low)**	1.75	(0.94-3.26)	0.079	**1.86**	(1.03-3.34)	**0.039**
NOXA1	Cytogenetically Normal	1	-	-	1	-	-
	t(15;17)	0.52	(0.17-1.62)	0.257	0.71	(0.25-2.01)	0.521
	t(8;21)	0.57	(0.11-3.03)	0.506	0.5	(0.1-2.57)	0.406
	inv(16)	0.34	(0.09-1.27)	0.109	0.33	(0.1-1.12)	0.076
	Intermediate Risk Cytogenetics	1.16	(0.54-2.5)	0.703	1.21	(0.57-2.58)	0.615
	Complex Cytogenetics	4.05	(1.65-9.96)	0.002	3.31	(1.44-7.6)	0.005
	Poor Risk Cytogenetics	1.76	(0.74-4.17)	0.203	3	(1.2-7.49)	0.018
	Age						
	***NOXA1* (High vs Low)**	0.74	(0.41-1.32)	0.31	0.7	(0.41-1.2)	0.195
NOXO1	Cytogenetically Normal	1	-	-	1	-	-
	t(15;17)	0.49	(0.16-1.53)	0.222	0.64	(0.23-1.78)	0.395
	t(8;21)	0.67	(0.13-3.54)	0.64	0.59	(0.12-2.97)	0.522
	inv(16)	0.41	(0.11-1.48)	0.174	0.42	(0.13-1.33)	0.14
	Intermediate Risk Cytogenetics	1.22	(0.57-2.64)	0.611	1.18	(0.56-2.49)	0.669
	Complex Cytogenetics	4.16	(1.67-10.34)	0.002	3.18	(1.39-7.26)	0.006
	Poor Risk Cytogenetics	2.09	(0.83-5.24)	0.116	2.97	(1.16-7.59)	0.023
	Age	1	-	-	1	-	-
	***NOXO1* (High vs Low)**	0.69	(0.4-1.22)	0.203	0.96	(0.57-1.62)	0.891

*Wald's p-value.

Age violated proportional hazard assumption in the TCGA dataset and was used as stratifier.

NOX3, NOX4, DUOX2 and NOXO1 were not expressed in GSE6891 dataset.

t(15;17) cytogenetic abnormality and NOXA1 expression violated proportional hazard assumption in GSE6891 dataset and were, therefore, excluded from the models.

Table S7. NOX expression according to molecular abnormalities with cytogenetically normal AML (CN-AML) samples in the 3 AML expression datasets.

		GSE6891 (CN-AML, N = 181)				GSE10358 (CN-AML, N = 92)			TCGA (CN-AML, N = 75)			
**Gene**	**Comparison**	**FC**	**p-value**	**q-value**		**FC**	**p-value**	**q-value**	**FC**	**p-value**	**q-value**	
NOX1	(NPM1 mt - FLT3ITD negative) vs the rest	0.02	0.1028	0.2056		-0.01	0.8666	0.9388	-0.12	0.519	0.613	
	(NPM1 mt - FLT3ITD positive) vs the rest	-0.01	0.1087	0.2174		0.05	0.7558	0.8932	0.25	0.3073	0.444	
	(NPM1 wt - FLT3ITD negative) vs the rest	0	0.6836	0.7889		0.01	0.8938	0.8938	-0.12	0.5386	0.797	
	(NPM1 wt - FLT3ITD positive) vs the rest	-0.01	0.0125	0.0417	*	-0.08	0.4635	0.6695	0.44	0.4316	0.808	
CYBB	(NPM1 mt - FLT3ITD negative) vs the rest	**1.62**	<0.001	0.002	**	0.4	0.4701	0.8037	0.78	0.1881	0.349	
	(NPM1 mt - FLT3ITD positive) vs the rest	-1	0.0522	0.174		0.03	0.9614	0.9614	-1.59	0.0745	0.275	
	(NPM1 wt - FLT3ITD negative) vs the rest	-0.64	0.2069	0.4138		0.06	0.8926	0.8938	0.55	0.2977	0.645	
	(NPM1 wt - FLT3ITD positive) vs the rest	0.34	0.5752	0.6247		-1.16	0.0741	0.1927	-2.09	0.27	0.808	
NOX3	(NPM1 mt - FLT3ITD negative) vs the rest	ND				0.05	0.1198	0.5522	0.01	0.9322	0.932	
	(NPM1 mt - FLT3ITD positive) vs the rest					-0.04	0.4556	0.8461	0.09	0.5579	0.725	
	(NPM1 wt - FLT3ITD negative) vs the rest					-0.02	0.5913	0.8938	-0.11	0.0353	0.115	
	(NPM1 wt - FLT3ITD positive) vs the rest					-0.02	0.6744	0.7457	0.25	0.4737	0.808	
NOX4	(NPM1 mt - FLT3ITD negative) vs the rest	ND				0	0.9659	0.9659	ND			
	(NPM1 mt - FLT3ITD positive) vs the rest					-0.11	0.0679	0.4268				
	(NPM1 wt - FLT3ITD negative) vs the rest					0.1	0.1212	0.3151				
	(NPM1 wt - FLT3ITD positive) vs the rest					-0.1	0.0444	0.1924				
NOX5	(NPM1 mt - FLT3ITD negative) vs the rest	0.01	0.8374	0.8374		-0.06	0.4574	0.8037	0.2	0.644	0.698	
	(NPM1 mt - FLT3ITD positive) vs the rest	-0.05	0.2067	0.3445		0.1	0.5433	0.8829	-0.06	0.908	0.908	
	(NPM1 wt - FLT3ITD negative) vs the rest	0.06	0.3477	0.5795		0.06	0.5322	0.8938	-0.12	0.7967	0.797	
	(NPM1 wt - FLT3ITD positive) vs the rest	-0.05	0.146	0.3482		-0.17	0.0191	0.1242	-0.26	0.6528	0.839	
DUOX1	(NPM1 mt - FLT3ITD negative) vs the rest	-0.02	0.6147	0.7684		-0.13	0.0111	0.1443	-0.38	0.075	0.217	
	(NPM1 mt - FLT3ITD positive) vs the rest	-0.01	0.8511	0.8511		0.11	0.2394	0.6224	-0.37	0.089	0.275	
	(NPM1 wt - FLT3ITD negative) vs the rest	0.01	0.7427	0.7889		0.11	0.0565	0.2448	0.6	0.0032	0.04	*
	(NPM1 wt - FLT3ITD positive) vs the rest	0.03	0.6247	0.6247		-0.16	0.1658	0.2694	0.01	0.9848	0.985	
DUOX2	(NPM1 mt - FLT3ITD negative) vs the rest	ND				-0.01	0.8308	0.9388	-0.42	0.1576	0.342	
	(NPM1 mt - FLT3ITD positive) vs the rest					0.04	0.7385	0.8932	-0.06	0.8377	0.908	
	(NPM1 wt - FLT3ITD negative) vs the rest					0.05	0.4616	0.8938	0.74	0.0326	0.115	
	(NPM1 wt - FLT3ITD positive) vs the rest					-0.18	0.0962	0.2084	-1.29	0.0019	0.025	*
CYBA	(NPM1 mt - FLT3ITD negative) vs the rest	0.22	0.1459	0.2432		-0.15	0.455	0.8037	0.14	0.443	0.576	
	(NPM1 mt - FLT3ITD positive) vs the rest	-0.09	0.5098	0.6373		0.08	0.7313	0.8932	-0.3	0.1581	0.275	
	(NPM1 wt - FLT3ITD negative) vs the rest	-0.22	0.1205	0.3012		0.05	0.7502	0.8938	0.05	0.7782	0.797	
	(NPM1 wt - FLT3ITD positive) vs the rest	0.23	0.1999	0.3482		0.08	0.6883	0.7457	-0.13	0.5688	0.822	
NCF1	(NPM1 mt - FLT3ITD negative) vs the rest	**1.89**	<0.001	<0.001	***	0.66	0.1699	0.5522	1.39	0.0208	0.208	

	(NPM1 mt - FLT3ITD positive) vs the rest	-0.16	0.6431	0.7146		0.81	0.0879	0.4268		-1.4	0.1406	0.275	
	(NPM1 wt - FLT3ITD negative) vs the rest	-0.83	0.0075	0.0375	*	-0.7	0.0521	0.2448		-0.23	0.6878	0.797	
	(NPM1 wt - FLT3ITD positive) vs the rest	**-1.55**	<0.001	0.001	***	-0.95	0.0702	0.1927		-1.91	0.1498	0.808	
NCF2	(NPM1 mt - FLT3ITD negative) vs the rest	**2.02**	<0.001	0.002	**	-0.14	0.8132	0.9388		0.54	0.3564	0.515	
	(NPM1 mt - FLT3ITD positive) vs the rest	-0.59	0.3314	0.4734		0.76	0.1723	0.56		-1.14	0.1605	0.275	
	(NPM1 wt - FLT3ITD negative) vs the rest	-1.46	0.0152	0.0507		-0.18	0.6978	0.8938		0.25	0.652	0.797	
	(NPM1 wt - FLT3ITD positive) vs the rest	0.43	0.5485	0.6247		-0.4	0.5267	0.6847		-0.71	0.4971	00808	
NCF4	(NPM1 mt - FLT3ITD negative) vs the rest	0.06	0.7389	0.821		-0.15	0.4946	0.8037		0.32	0.0555	0.217	
	(NPM1 mt - FLT3ITD positive) vs the rest	0.48	<0.001	0.009	**	0.53	0.0985	0.4268		0.35	0.0448	0.275	
	(NPM1 wt - FLT3ITD negative) vs the rest	-0.63	0.0071	0.0375	*	-0.46	0.0229	0.2448		-0.54	0.0061	0.04	*
	(NPM1 wt - FLT3ITD positive) vs the rest	0.21	0.2089	0.3482		0.8	<0.001	<0.001	***	-0.01	0.9725	0.985	
NOXA1	(NPM1 mt - FLT3ITD negative) vs the rest	0.11	0.0198	0.0495	*	0.14	0.1697	0.5522		0.85	0.032	0.208	
	(NPM1 mt - FLT3ITD positive) vs the rest	-0.07	0.0298	0.149		0.14	0.3248	0.7037		-0.72	0.1695	0.275	
	(NPM1 wt - FLT3ITD negative) vs the rest	0.01	0.7889	0.7889		-0.19	0.0796	0.2587		-0.25	0.5736	0.797	
	(NPM1 wt - FLT3ITD positive) vs the rest	-0.09	<0.001	0.0035	**	-0.02	0.8413	0.8413		-1.03	0.2061	0.808	
NOXO1	(NPM1 mt - FLT3ITD negative) vs the rest	ND				-0.04	0.6166	0.8906		0.25	0.251	0.408	
	(NPM1 mt - FLT3ITD positive) vs the rest					0.01	0.9067	0.9614		0.11	0.7188	0.85	
	(NPM1 wt - FLT3ITD negative) vs the rest					-0.01	0.8361	0.8938		-0.29	0.1547	0.402	
	(NPM1 wt - FLT3ITD positive) vs the rest					0.11	0.1414	0.2626		-0.19	0.7095	0.839	

Student's t-test was used for pairwise comparisons. q-values: adjusted p-values calculated using the BH method.

NOX4, DUOX2, and NOXO1 were excluded for GSE6891 dataset because they are mostly not expressed. NOX4 was also excluded from the TCGA dataset for the same reason.

Table S8. Univariate cox regression analysis of NOX in the cytogenetically normal (CN-AML) subset of 3 AML datasets.

A) OS table

	**GSE6891 (CN-AML, OS N = 122)**			**GSE10385 (CN-AML, OS N = 93)**			**TCGA (CN-AML, OS N = 75)**		
**Variable**	**Hazard Ratio**	**95% CI**	**Wald's p-value**	**Hazard Ratio**	**95% CI**	**Wald's p-value**	**Hazard Ratio**	**95% CI**	**Wald's p-value**
NOX1 (High vs Low)	0.65	(0.28-1.5)	0.316	1.09	(0.63-1.89)	0.758	1.12	(0.65-1.95)	0.68
CYBB (High vs Low)	1.31	(0.83-2.08)	0.244	**1.82**	**(1.05-3.16)**	**0.032**	1.35	(0.78-2.34)	0.29
NOX3 (High vs Low)	-	-	-	0.6	(0.35-1.02)	0.061	1.1	(0.34-3.57)	0.869
NOX4 (High vs Low)	-	-	-	1.24	(0.72-2.11)	0.437	-	-	-
NOX5 (High vs Low)	1	(0.48-2.08)	0.996	0.62	(0.36-1.07)	0.084	0.95	(0.55-1.66)	0.865
DUOX1 (High vs Low)	0.96	(0.55-1.68)	0.895	0.99	(0.58-1.69)	0.971	0.63	(0.36-1.09)	0.099
DUOX2 (High vs Low)	-	-	-	1.16	(0.68-1.97)	0.596	1.11	(0.64-1.93)	0.716
CYBA (High vs Low)	0.94	(0.59-1.48)	0.777	0.95	(0.55-1.63)	0.85	1.18	(0.68-2.06)	0.548
NCF1 (High vs Low)	1.16	(0.73-1.83)	0.531	1.93	(1.11-3.36)	0.02	1.47	(0.84-2.57)	0.176
NCF2 (High vs Low)	1.13	(0.71-1.79)	0.601	1.66	(0.96-2.86)	0.068	1.5	(0.85-2.63)	0.161
NCF4 (High vs Low)	1.19	(0.75-1.89)	0.458	1.88	(1.08-3.27)	0.025	1.33	(0.76-2.3)	0.318
NOXA1 (High vs Low)	0.88	(0.52-1.49)	0.638	0.81	(0.47-1.38)	0.432	0.75	(0.43-1.3)	0.31
NOXO1 (High vs Low)	-	-	-	0.7	(0.41-1.2)	0.192	1	(0.58-1.73)	0.993

B) EFS table

	**GSE6891 (CN-AML, EFS N = 122)**			**GSE10385 (CN-AML, EFS N = 93)**			**TCGA (CN-AML, EFS N = 75)**		
**Variable**	**Hazard Ratio**	**95% CI**	**Wald's p-value**	**Hazard Ratio**	**95% CI**	**Wald's p-value**	**Hazard Ratio**	**95% CI**	**Wald's p-value**
NOX1 (High vs Low)	0.54	(0.23-1.24)	0.144	1.08	(0.66-1.77)	0.758	1.14	(0.69-1.89)	0.604
CYBB (High vs Low)	1.16	(0.75-1.8)	0.51	1.49	(0.91-2.44)	0.109	1.1	(0.66-1.82)	0.719
NOX3 (High vs Low)	-	-	-	0.67	(0.41-1.08)	0.101	1	(0.36-2.78)	0.994
NOX4 (High vs Low)	-	-	-	1.13	(0.7-1.84)	0.617	-	-	-
NOX5 (High vs Low)	0.93	(0.45-1.93)	0.844	0.73	(0.45-1.19)	0.207	1.09	(0.66-1.83)	0.729
DUOX1 (High vs Low)	0.77	(0.45-1.33)	0.352	1.03	(0.63-1.67)	0.911	0.69	(0.42-1.14)	0.15
DUOX2 (High vs Low)	-	-	-	1.08	(0.66-1.76)	0.756	1.05	(0.64-1.74)	0.839
CYBA (High vs Low)	0.8	(0.51-1.24)	0.314	0.9	(0.56-1.47)	0.687	0.97	(0.59-1.61)	0.921
NCF1 (High vs Low)	0.94	(0.61-1.45)	0.772	**1.88**	(1.14-3.11)	**0.014**	1.16	(0.7-1.93)	0.56
NCF2 (High vs Low)	0.92	(0.59-1.42)	0.703	1.39	(0.85-2.27)	0.184	1.24	(0.74-2.05)	0.415
NCF4 (High vs Low)	-	-	-	**1.99**	(1.2-3.29)	**0.008**	1.39	(0.84-2.31)	0.201
NOXA1 (High vs Low)	1.02	(0.62-1.66)	0.943	0.84	(0.52-1.37)	0.494	0.83	(0.5-1.39)	0.48
NOXO1 (High vs Low)	-	-	-	0.72	(0.44-1.17)	0.182	1.09	(0.66-1.81)	0.733

NOX3, NOX4, DUOX2 and NOXO1 were not expressed in this dataset; NOX4 was not expressed in the TCGA dataset.
NCF4 violated proportional hazard's assumption for EFS of CN-AML samples in the GSE6891 dataset.

Table S9. Cox regression analysis of NOX in the cytogenetically normal subset (CN-AML) of 3 AML datasets after adjustment for age and NPM1/FLT3 status.

**A) GSE6891 (CN-AML, N = 122)**		**GSE6891 (OS N = 122)**			**GSE6891 (EFS N = 122)**		
**Multivariate Model**	**Variable**	**Hazard Ratio**	**95% CI**	**P***	**Hazard Ratio**	**95% CI**	**P***
NOX1	NPM1 mt - FLT3ITD negative	1	-	-	1	-	-
	NPM1 mt - FLT3ITD positive	1.96	(0.99-3.89)	0.052	1.84	(0.96-3.51)	0.065
	NPM1 wt - FLT3ITD negative	1.54	(0.78-3.02)	0.212	1.36	(0.71-2.58)	0.35
	NPM1 wt - FLT3ITD positive	3.39	(1.55-7.42)	0.002	3.25	(1.54-6.87)	0.002
	Age	1.01	(0.99-1.03)	0.298	1.01	(0.99-1.03)	0.509
	NOX1 (High vs Low)	0.78	(0.33-1.85)	0.581	0.65	(0.28-1.52)	0.321
CYBB	NPM1 mt - FLT3ITD negative	1	-	-	1	-	-
	NPM1 mt - FLT3ITD positive	2.24	(1.12-4.49)	0.022	2.03	(1.05-3.9)	0.035
	NPM1 wt - FLT3ITD negative	1.69	(0.85-3.35)	0.131	1.46	(0.76-2.8)	0.253
	NPM1 wt - FLT3ITD positive	3.54	(1.63-7.69)	0.001	3.43	(1.63-7.2)	0.001
	Age	1.01	(0.99-1.03)	0.399	1	(0.98-1.02)	0.66
	CYBB (High vs Low)	1.39	(0.86-2.24)	0.183	1.16	(0.73-1.86)	0.524
NOX3	NPM1 mt - FLT3ITD negative	NA			NA		
	NPM1 mt - FLT3ITD positive						
	NPM1 wt - FLT3ITD negative						
	NPM1 wt - FLT3ITD positive						
	Age						
	NOX3 (High vs Low)						
NOX4	NPM1 mt - FLT3ITD negative	NA			NA		
	NPM1 mt - FLT3ITD positive						
	NPM1 wt - FLT3ITD negative						
	NPM1 wt - FLT3ITD positive						
	Age						
	NOX4 (High vs Low)						
NOX5	NPM1 mt - FLT3ITD negative	1	-	-	1	-	-
	NPM1 mt - FLT3ITD positive	2.02	(1.03-3.96)	0.042	1.94	(1.02-3.69)	0.044
	NPM1 wt - FLT3ITD negative	1.58	(0.8-3.1)	0.184	1.41	(0.74-2.68)	0.29
	NPM1 wt - FLT3ITD positive	3.57	(1.64-7.79)	0.001	3.49	(1.66-7.33)	0.001
	Age	1.01	(0.99-1.03)	0.312	1.01	(0.99-1.03)	0.575
	NOX5 (High vs Low)	0.91	(0.42-1.95)	0.803	0.96	(0.45-2.03)	0.913
DUOX1	NPM1 mt - FLT3ITD negative	1	-	-	1	-	-
	NPM1 mt - FLT3ITD positive	2.03	(1.03-3.98)	0.04	1.95	(1.03-3.69)	0.042

	NPM1 wt - FLT3ITD negative	1.57	(0.8-3.08)	0.192	1.44	(0.76-2.73)	0.266
	NPM1 wt - FLT3ITD positive	3.53	(1.63-7.67)	0.001	3.45	(1.64-7.26)	0.001
	Age	1.01	(0.99-1.03)	0.326	1.01	(0.99-1.03)	0.568
	DUOX1 (High vs Low)	1.02	(0.58-1.78)	0.957	0.81	(0.46-1.4)	0.442
DUOX2	NPM1 mt - FLT3ITD negative	NA			NA		
	NPM1 mt - FLT3ITD positive						
	NPM1 wt - FLT3ITD negative						
	NPM1 wt - FLT3ITD positive						
	Age						
	DUOX2 (High vs Low)						
CYBA	NPM1 mt - FLT3ITD negative	1	-	-	1	-	-
	NPM1 mt - FLT3ITD positive	2.02	(1.03-3.97)	0.04	1.98	(1.04-3.76)	0.037
	NPM1 wt - FLT3ITD negative	1.57	(0.8-3.07)	0.192	1.4	(0.74-2.67)	0.301
	NPM1 wt - FLT3ITD positive	3.56	(1.64-7.7)	0.001	3.72	(1.77-7.81)	0.001
	Age	1.01	(0.99-1.03)	0.322	1.01	(0.99-1.03)	0.514
	CYBA (High vs Low)	0.9	(0.57-1.42)	0.644	0.72	(0.46-1.13)	0.15
NCF1	NPM1 mt - FLT3ITD negative	1	-	-	1	-	-
	NPM1 mt - FLT3ITD positive	2.13	(1.08-4.22)	0.03	1.93	(1.01-3.69)	0.046
	NPM1 wt - FLT3ITD negative	1.67	(0.84-3.3)	0.143	1.4	(0.73-2.68)	0.313
	NPM1 wt - FLT3ITD positive	3.69	(1.69-8.04)	0.001	3.47	(1.65-7.3)	0.001
	Age	1.01	(0.99-1.03)	0.365	1.01	(0.99-1.03)	0.573
	NCF1 (High vs Low)	1.26	(0.79-2.01)	0.332	0.97	(0.62-1.52)	0.887
NCF2	NPM1 mt - FLT3ITD negative	1	-	-	1	-	-
	NPM1 mt - FLT3ITD positive	2.08	(1.05-4.11)	0.035	1.91	(1-3.64)	0.049
	NPM1 wt - FLT3ITD negative	1.63	(0.82-3.23)	0.162	1.37	(0.71-2.62)	0.346
	NPM1 wt - FLT3ITD positive	3.56	(1.64-7.72)	0.001	3.5	(1.67-7.35)	0.001
	Age	1.01	(0.99-1.03)	0.35	1.01	(0.99-1.03)	0.542
	NCF2 (High vs Low)	1.15	(0.72-1.85)	0.555	0.89	(0.56-1.4)	0.61
NCF4	NPM1 mt - FLT3ITD negative	1	-	-	NA		
	NPM1 mt - FLT3ITD positive	2.06	(1.05-4.06)	0.036			
	NPM1 wt - FLT3ITD negative	1.64	(0.83-3.23)	0.153			
	NPM1 wt - FLT3ITD positive	3.77	(1.71-8.28)	0.001			
	Age	1.01	(0.99-1.03)	0.272			
	NCF4 (High vs Low)	1.29	(0.81-2.07)	0.283			
NOXA1	NPM1 mt - FLT3ITD negative	1	-	-	1	-	-
	NPM1 mt - FLT3ITD positive	2.02	(1.03-3.99)	0.042	1.99	(1.04-3.83)	0.038
	NPM1 wt - FLT3ITD negative	1.56	(0.79-3.09)	0.197	1.45	(0.76-2.76)	0.266

	NPM1 wt - FLT3ITD positive	3.52	(1.61-7.68)	0.002	3.55	(1.69-7.46)	0.001
	Age	1.01	(0.99-1.03)	0.326	1.01	(0.99-1.03)	0.599
	NOXA1 (High vs Low)	0.98	(0.58-1.68)	0.953	1.13	(0.68-1.86)	0.638
NOXO1	NPM1 mt - FLT3ITD negative	NA			NA		
	NPM1 mt - FLT3ITD positive						
	NPM1 wt - FLT3ITD negative						
	NPM1 wt - FLT3ITD positive						
	Age						
	NOXO1 (High vs Low)						

**B) GSE10385 (CN-AML, N = 92)**		**GSE10385 (OS N = 92)**			**GSE10385 (EFS N = 92)**		
**Multivariate Model**	**Variable**	**Hazard Ratio**	**95% CI**	**P***	**Hazard Ratio**	**95% CI**	**P***
NOX1	NPM1 mt - FLT3ITD negative	1	-	-	1	-	-
	NPM1 mt - FLT3ITD positive	2.47	(1.17-5.19)	0.017	2.94	(1.45-5.95)	0.003
	NPM1 wt - FLT3ITD negative	0.58	(0.29-1.18)	0.132	0.84	(0.45-1.57)	0.594
	NPM1 wt - FLT3ITD positive	3.14	(1.29-7.64)	0.012	2.78	(1.17-6.66)	0.021
	Age	1.03	(1.01-1.05)	0.007	1.02	(1.01-1.04)	0.005
	NOX1 (High vs Low)	1.3	(0.72-2.34)	0.385	1.21	(0.72-2.04)	0.477
CYBB	NPM1 mt - FLT3ITD negative	1	-	-	1	-	-
	NPM1 mt - FLT3ITD positive	2.45	(1.17-5.12)	0.017	2.92	(1.44-5.9)	0.003
	NPM1 wt - FLT3ITD negative	0.62	(0.31-1.24)	0.176	0.89	(0.48-1.64)	0.707
	NPM1 wt - FLT3ITD positive	4.34	(1.73-10.85)	0.002	3.51	(1.45-8.52)	0.005
	Age	1.02	(1-1.04)	0.063	1.02	(1-1.04)	0.025
	CYBB (High vs Low)	**2.07**	(1.12-3.84)	**0.021**	1.54	(0.9-2.63)	0.118
NOX3	NPM1 mt - FLT3ITD negative	1	-	-	1	-	-
	NPM1 mt - FLT3ITD positive	1.96	(0.89-4.32)	0.094	2.57	(1.22-5.39)	0.013
	NPM1 wt - FLT3ITD negative	0.55	(0.27-1.13)	0.102	0.83	(0.44-1.55)	0.555
	NPM1 wt - FLT3ITD positive	2.9	(1.17-7.18)	0.021	2.73	(1.14-6.54)	0.024
	Age	1.03	(1.01-1.05)	0.008	1.02	(1.01-1.04)	0.006
	NOX3 (High vs Low)	0.67	(0.37-1.21)	0.184	0.78	(0.46-1.3)	0.337
NOX4	NPM1 mt - FLT3ITD negative	1	-	-	1	-	-
	NPM1 mt - FLT3ITD positive	2.49	(1.19-5.21)	0.016	3.07	(1.51-6.23)	0.002
	NPM1 wt - FLT3ITD negative	0.61	(0.31-1.23)	0.168	0.91	(0.49-1.69)	0.771
	NPM1 wt - FLT3ITD positive	3.59	(1.48-8.71)	0.005	3.19	(1.34-7.57)	0.009
	Age	1.03	(1.01-1.05)	0.006	1.02	(1.01-1.04)	0.004
	NOX4 (High vs Low)	1.53	(0.89-2.64)	0.128	1.37	(0.83-2.25)	0.214

NOX5	NPM1 mt - FLT3ITD negative	1	-	-	1	-	-
	NPM1 mt - FLT3ITD positive	2.36	(1.13-4.94)	0.023	2.95	(1.46-5.98)	0.003
	NPM1 wt - FLT3ITD negative	0.64	(0.32-1.29)	0.213	0.92	(0.5-1.71)	0.796
	NPM1 wt - FLT3ITD positive	3.28	(1.37-7.86)	0.008	2.95	(1.26-6.91)	0.013
	Age	1.02	(1-1.04)	0.022	1.02	(1-1.04)	0.013
	NOX5 (High vs Low)	0.71	(0.41-1.26)	0.243	0.78	(0.47-1.31)	0.352
DUOX1	NPM1 mt - FLT3ITD negative	1	-	-	1	-	-
	NPM1 mt - FLT3ITD positive	2.4	(1.1-5.23)	0.028	2.95	(1.41-6.17)	0.004
	NPM1 wt - FLT3ITD negative	0.62	(0.3-1.26)	0.187	0.89	(0.47-1.69)	0.73
	NPM1 wt - FLT3ITD positive	3.37	(1.39-8.16)	0.007	3	(1.27-7.08)	0.012
	Age	1.03	(1.01-1.05)	0.014	1.02	(1.01-1.04)	0.009
	DUOX1 (High vs Low)	0.97	(0.54-1.74)	0.92	0.95	(0.56-1.6)	0.84
DUOX2	NPM1 mt - FLT3ITD negative	1	-	-	1	-	-
	NPM1 mt - FLT3ITD positive	2.58	(1.22-5.46)	0.013	3.03	(1.49-6.18)	0.002
	NPM1 wt - FLT3ITD negative	0.59	(0.3-1.19)	0.14	0.87	(0.47-1.6)	0.651
	NPM1 wt - FLT3ITD positive	3.52	(1.44-8.59)	0.006	3.03	(1.28-7.15)	0.012
	Age	1.03	(1.01-1.05)	0.009	1.02	(1.01-1.04)	0.007
	DUOX2 (High vs Low)	1.42	(0.81-2.47)	0.217	1.23	(0.74-2.03)	0.426
CYBA	NPM1 mt - FLT3ITD negative	1	-	-	1	-	-
	NPM1 mt - FLT3ITD positive	2.4	(1.14-5.04)	0.021	2.88	(1.43-5.83)	0.003
	NPM1 wt - FLT3ITD negative	0.61	(0.31-1.22)	0.161	0.88	(0.48-1.62)	0.677
	NPM1 wt - FLT3ITD positive	3.46	(1.4-8.51)	0.007	2.96	(1.23-7.07)	0.015
	Age	1.03	(1.01-1.05)	0.013	1.02	(1.01-1.04)	0.007
	CYBA (High vs Low)	1.09	(0.62-1.94)	0.763	0.99	(0.6-1.63)	0.957
NCF1	NPM1 mt - FLT3ITD negative	1	-	-	1	-	-
	NPM1 mt - FLT3ITD positive	2.61	(1.24-5.48)	0.012	3.11	(1.53-6.32)	0.002
	NPM1 wt - FLT3ITD negative	0.67	(0.34-1.35)	0.264	0.99	(0.53-1.83)	0.974
	NPM1 wt - FLT3ITD positive	4.34	(1.73-10.91)	0.002	3.83	(1.57-9.35)	0.003
	Age	1.02	(1-1.04)	0.042	1.02	(1-1.04)	0.033
	NCF1 (High vs Low)	**1.97**	(1.08-3.59)	**0.027**	**1.87**	(1.08-3.22)	**0.024**
NCF2	NPM1 mt - FLT3ITD negative	1	-	-	1	-	-
	NPM1 mt - FLT3ITD positive	2.66	(1.26-5.62)	0.01	3.05	(1.5-6.19)	0.002
	NPM1 wt - FLT3ITD negative	0.66	(0.33-1.32)	0.244	0.93	(0.5-1.72)	0.822
	NPM1 wt - FLT3ITD positive	4.17	(1.67-10.42)	0.002	3.43	(1.42-8.3)	0.006
	Age	1.02	(1-1.04)	0.032	1.02	(1-1.04)	0.014

	NCF2 (High vs Low)	**1.81**	(1.01-3.24)	**0.047**	1.44	(0.86-2.41)	0.166
NCF4	NPM1 mt - FLT3ITD negative	1	-	-	1	-	-
	NPM1 mt - FLT3ITD positive	2.35	(1.12-4.92)	0.023	2.86	(1.41-5.79)	0.004
	NPM1 wt - FLT3ITD negative	0.66	(0.33-1.34)	0.254	1	(0.53-1.86)	0.992
	NPM1 wt - FLT3ITD positive	2.95	(1.19-7.32)	0.02	2.46	(1.03-5.9)	0.044
	Age	1.03	(1.01-1.05)	0.012	1.02	(1.01-1.04)	0.007
	NCF4 (High vs Low)	1.39	(0.76-2.56)	0.287	1.7	(0.98-2.95)	0.061
NOXA1	NPM1 mt - FLT3ITD negative	1	-	-	1	-	-
	NPM1 mt - FLT3ITD positive	2.62	(1.23-5.6)	0.013	3	(1.47-6.11)	0.002
	NPM1 wt - FLT3ITD negative	0.56	(0.28-1.13)	0.105	0.84	(0.45-1.57)	0.585
	NPM1 wt - FLT3ITD positive	2.95	(1.2-7.24)	0.018	2.74	(1.14-6.58)	0.024
	Age	1.02	(1-1.04)	0.015	1.02	(1.01-1.04)	0.009
	NOXA1 (High vs Low)	0.67	(0.37-1.22)	0.19	0.81	(0.48-1.37)	0.43
NOXO1	NPM1 mt - FLT3ITD negative	1	-	-	1	-	-
	NPM1 mt - FLT3ITD positive	2.7	(1.28-5.7)	0.009	3.13	(1.54-6.35)	0.002
	NPM1 wt - FLT3ITD negative	0.59	(0.3-1.18)	0.136	0.84	(0.46-1.56)	0.588
	NPM1 wt - FLT3ITD positive	4.44	(1.76-11.18)	0.002	3.61	(1.49-8.7)	0.004
	Age	1.03	(1.01-1.05)	0.011	1.02	(1.01-1.04)	0.007
	NOXO1 (High vs Low)	**0.51**	(0.29-0.91)	**0.022**	**0.59**	(0.36-0.99)	**0.044**

**C) TCGA (CN-AML, N = 75)**		**TCGA OS (CN-AML, N = 75)**			**TCGA EFS (CN-AML, N = 75)**		
**Multivariate Model**	**Variable**	**Hazard Ratio**	**95% CI**	**P***	**Hazard Ratio**	**95% CI**	**P***
NOX1	NPM1 mt - FLT3ITD negative	1	-	-	1	-	-
	NPM1 mt - FLT3ITD positive	1.97	(0.42-9.19)	0.386	1.42	(0.37-5.45)	0.612
	NPM1 wt - FLT3ITD negative	0.41	(0.12-1.47)	0.172	0.43	(0.14-1.3)	0.134
	NPM1 wt - FLT3ITD positive	33255926.79	(0-Inf)	0.998	0.43	(0.02-8.46)	0.576
	Age	stratifier	stratifier	stratifier	stratifier	stratifier	stratifier
	NOX1 (High vs Low)	1.16	(0.43-3.18)	0.769	0.67	(0.25-1.77)	0.416
CYBB	NPM1 mt - FLT3ITD negative	1	-	-	1	-	-
	NPM1 mt - FLT3ITD positive	1.58	(0.31-8.08)	0.584	1.24	(0.32-4.85)	0.761
	NPM1 wt - FLT3ITD negative	0.36	(0.1-1.37)	0.134	0.45	(0.15-1.35)	0.156
	NPM1 wt - FLT3ITD positive	15611539.72	(0-Inf)	0.999	0.4	(0.02-8.64)	0.563
	Age	stratifier	stratifier	stratifier	stratifier	stratifier	stratifier
	CYBB (High vs Low)	0.53	(0.14-2.07)	0.363	0.8	(0.25-2.53)	0.706
NOX3	NPM1 mt - FLT3ITD negative	1	-	-	1	-	-

	NPM1 mt - FLT3ITD positive	2.43	(0.44-13.53)	0.311	1.32	(0.35-4.95)	0.682
	NPM1 wt - FLT3ITD negative	0.46	(0.13-1.71)	0.248	0.45	(0.15-1.37)	0.161
	NPM1 wt - FLT3ITD positive	37388201.53	(0-Inf)	0.998	0.49	(0.02-10.72)	0.648
	Age	stratifier	stratifier	stratifier	stratifier	stratifier	stratifier
	NOX3 (High vs Low)	5.98	(0.39-91.47)	0.199	0.88	(0.11-6.97)	0.9
NOX4	NPM1 mt - FLT3ITD negative	NA			NA		
	NPM1 mt - FLT3ITD positive						
	NPM1 wt - FLT3ITD negative						
	NPM1 wt - FLT3ITD positive						
	Age						
	NOX4 (High vs Low)						
NOX5	NPM1 mt - FLT3ITD negative	1	-	-	1	-	-
	NPM1 mt - FLT3ITD positive	2.1	(0.45-9.87)	0.346	1.32	(0.35-4.96)	0.683
	NPM1 wt - FLT3ITD negative	0.39	(0.11-1.43)	0.154	0.46	(0.16-1.38)	0.168
	NPM1 wt - FLT3ITD positive	51167069.23	(0-Inf)	0.998	0.46	(0.02-9.13)	0.613
	Age	stratifier	stratifier	stratifier	stratifier	stratifier	stratifier
	NOX5 (High vs Low)	0.61	(0.17-2.24)	0.457	1.06	(0.36-3.12)	0.918
DUOX1	NPM1 mt - FLT3ITD negative	1	-	-	1	-	-
	NPM1 mt - FLT3ITD positive	2.17	(0.45-10.52)	0.338	1.68	(0.41-6.93)	0.473
	NPM1 wt - FLT3ITD negative	0.44	(0.12-1.65)	0.225	0.53	(0.17-1.61)	0.261
	NPM1 wt - FLT3ITD positive	30070994.27	(0-Inf)	0.998	0.53	(0.02-11.48)	0.684
	Age	stratifier	stratifier	stratifier	stratifier	stratifier	stratifier
	DUOX1 (High vs Low)	0.85	(0.29-2.43)	0.756	0.59	(0.22-1.56)	0.284
DUOX2	NPM1 mt - FLT3ITD negative	1	-	-	1	-	-
	NPM1 mt - FLT3ITD positive	2.63	(0.47-14.8)	0.272	1.78	(0.41-7.76)	0.441
	NPM1 wt - FLT3ITD negative	0.46	(0.12-1.67)	0.235	0.47	(0.16-1.41)	0.176
	NPM1 wt - FLT3ITD positive	24214322.93	(0-Inf)	0.998	0.27	(0.01-6.23)	0.41
	Age	stratifier	stratifier	stratifier	stratifier	stratifier	stratifier
	DUOX2 (High vs Low)	0.66	(0.21-2.05)	0.474	0.57	(0.2-1.6)	0.285
CYBA	NPM1 mt - FLT3ITD negative	1	-	-	1	-	-
	NPM1 mt - FLT3ITD positive	2.07	(0.45-9.65)	0.352	1.33	(0.35-5)	0.674
	NPM1 wt - FLT3ITD negative	0.41	(0.12-1.47)	0.173	0.46	(0.15-1.37)	0.161
	NPM1 wt - FLT3ITD positive	33364906.53	(0-Inf)	0.998	0.45	(0.02-9.4)	0.606

	Age	stratifier	stratifier	stratifier	stratifier	stratifier	stratifier
	CYBA (High vs Low)	1.1	(0.37-3.31)	0.863	1.04	(0.38-2.87)	0.937
NCF1	NPM1 mt - FLT3ITD negative	1	-	-	1	-	-
	NPM1 mt - FLT3ITD positive	1.94	(0.39-9.74)	0.419	1.36	(0.34-5.41)	0.66
	NPM1 wt - FLT3ITD negative	0.41	(0.11-1.49)	0.173	0.46	(0.16-1.37)	0.165
	NPM1 wt - FLT3ITD positive	28842089.93	(0-Inf)	0.998	0.48	(0.02-10.11)	0.639
	Age	stratifier	stratifier	stratifier	stratifier	stratifier	stratifier
	NCF1 (High vs Low)	0.88	(0.24-3.25)	0.853	1.09	(0.36-3.29)	0.885
NCF2	NPM1 mt - FLT3ITD negative	1	-	-	1	-	-
	NPM1 mt - FLT3ITD positive	1.81	(0.36-9.04)	0.471	1.33	(0.34-5.17)	0.683
	NPM1 wt - FLT3ITD negative	0.4	(0.11-1.45)	0.164	0.46	(0.16-1.36)	0.162
	NPM1 wt - FLT3ITD positive	23357611.36	(0-Inf)	0.998	0.46	(0.02-9.63)	0.619
	Age	stratifier	stratifier	stratifier	stratifier	stratifier	stratifier
	NCF2 (High vs Low)	0.72	(0.19-2.8)	0.638	1.01	(0.31-3.26)	0.985
NCF4	NPM1 mt - FLT3ITD negative	1	-	-	1	-	-
	NPM1 mt - FLT3ITD positive	2.05	(0.44-9.5)	0.36	1.32	(0.35-4.98)	0.683
	NPM1 wt - FLT3ITD negative	0.42	(0.12-1.5)	0.181	0.47	(0.16-1.4)	0.174
	NPM1 wt - FLT3ITD positive	27276898.34	(0-Inf)	0.998	0.47	(0.02-9.31)	0.62
	Age	stratifier	stratifier	stratifier	stratifier	stratifier	stratifier
	NCF4 (High vs Low)	1.24	(0.35-4.37)	0.735	1.17	(0.42-3.26)	0.76
NOXA1	NPM1 mt - FLT3ITD negative	1	-	-	1	-	-
	NPM1 mt - FLT3ITD positive	2.48	(0.49-12.4)	0.27	1.53	(0.39-6)	0.542
	NPM1 wt - FLT3ITD negative	0.44	(0.12-1.62)	0.217	0.47	(0.16-1.43)	0.185
	NPM1 wt - FLT3ITD positive	35300663.96	(0-Inf)	0.998	0.66	(0.03-14.26)	0.791
	Age	stratifier	stratifier	stratifier	stratifier	stratifier	stratifier
	NOXA1 (High vs Low)	0.43	(0.13-1.37)	0.152	0.51	(0.17-1.51)	0.226
NOXO1	NPM1 mt - FLT3ITD negative	1	-	-	1	-	-
	NPM1 mt - FLT3ITD positive	2.14	(0.45-10.15)	0.336	1.32	(0.35-5.04)	0.685
	NPM1 wt - FLT3ITD negative	0.42	(0.12-1.5)	0.183	0.46	(0.16-1.37)	0.162
	NPM1 wt - FLT3ITD positive	33876343.57	(0-Inf)	0.998	0.46	(0.02-9.38)	0.616
	Age	stratifier	stratifier	stratifier	stratifier	stratifier	stratifier
	NOXO1 (High vs Low)	0.84	(0.3-2.31)	0.733	1.01	(0.38-2.71)	0.977

*Wald's p-value

Table S10. Distribution of FAB subtypes within cytogenetic groups in AML.

Dataset	FAB	[n (%)]							
		all	Normal karyotype	t(8;21)	t(15;17)	inv(16)	Intermediate Risk Cytogenetics	Complex Cytogenetics	Poor Risk Cytogenetics
GSE6891	**M0**	14 (3.54%)	3 (1.7%)	-	-	-	6 (8.11%)	3 (25%)	2 (4.55%)
	**M1**	87 (22%)	52 (29.5%)	2 (5.71%)	-	2 (5.88%)	18 (24.3%)	1 (8.33%)	12 (27.3%)
	**M2**	96 (24.24%)	36 (20.5%)	**30 (85.7%)**	-	3 (8.82%)	16 (21.6%)	3 (25%)	8 (18.2%)
	**M3**	24 (6.1%)	2 (1.14%)	-	**21 (100%)**	-	1 (1.35%)	-	-
	**M4**	81 (20.5%)	28 (15.9%)	3 (8.57%)	-	**26 (76.5%)**	14 (18.9%)	4 (33.3%)	6 (13.6%)
	**M5**	90 (22.7%)	52 (29.5%)	-	-	3 (8.82%)	18 (24.3%)	1 (8.33%)	16 (36.4%)
	**M6**	4 (1.01%)	3 (1.7%)	-	-	-	1 (1.35%)	-	-
GSE10385	**M0**	16 (7.27%)	5 (5.38%)	-	-	-	3 (8.11%)	4 (25%)	4 (26.7%)
	**M1**	40 (18.2%)	24 (25.8%)	2 (16.7%)	-	-	10 (27%)	2 (12.5%)	2 (13.3%)
	**M2**	55 (25%)	29 (31.2%)	**10 (83.3%)**	-	1 (6.25%)	8 (21.6%)	4 (25%)	3 (20%)
	**M3**	33 (15%)	2 (2.15%)	-	**31 (100%)**	-	-	-	-
	**M4**	56 (25.5%)	23 (24.7%)	-	-	**15 (93.8%)**	11 (29.7%)	2 (12.5%)	5 (33.3%)
	**M5**	16 (7.27%)	10 (10.8%)	-	-	-	4 (10.8%)	1 (6.25%)	1 (6.67%)
	**M6**	1 (0.455%)	-	-	-	-	-	1 (6.25%)	-
	**M7**	3 (1.36%)	-	-	-	-	1 (2.7%)	2 (12.5%)	-
TCGA	**M0**	16 (9.52%)	3 (4.05%)	-	-	-	4 (19%)	7 (31.8%)	2 (11.1%)
	**M1**	43 (25.6%)	24 (32.4%)	2 (28.6%)	-	2 (20%)	5 (23.8%)	5 (22.7%)	5 (27.8%)
	**M2**	36 (21.4%)	19 (25.7%)	**5 (71.4%)**	-	1 (10%)	3 (14.3%)	4 (18.2%)	4 (22.2%)
	**M3**	16 (9.52%)	-	-	**16 (100%)**	-	-	-	-
	**M4**	34 (20.2%)	15 (20.3%)	-	-	**7 (70%)**	7 (33.3%)	2 (9.09%)	3 (16.7%)
	**M5**	18 (10.7%)	12 (16.2%)	-	-	-	1 (4.76%)	1 (4.55%)	4 (22.2%)
	**M6**	2 (1.19%)	-	-	-	-	1 (4.76%)	1 (4.55%)	-
	**M7**	3 (1.79%)	1 (1.35%)	-	-	-	-	2 (9.09%)	-
